# Examining global Indigenous community wellness worker models: a rapid review

**DOI:** 10.1186/s12939-024-02185-5

**Published:** 2024-05-02

**Authors:** Kayla M. Fitzpatrick, Erynne Sjoblom, Giulia Puinean, Heath Robson, Sandra M. Campbell, Bryan Fayant, Stephanie Montesanti

**Affiliations:** 1 School of Public Health, University of Alberta, 3-300 Eddmonton Clinic Health Academy, Edmonton, AB T6G 1C9 Canada; 2 John W. Scott Health Sciences Library, Mackenzie Health Science Centre, University of Alberta, 8440 - 112 St, Edmonton, AB T6G 2B7 Canada; 3 McMurray Métis Local 1935, 441 Sakitawaw Trail, Fort McMurray, AB AB T9H 4P3 Canada; 4 Centre for Healthy Communities, School of Public Health, University of Alberta, Edmonton, AB Canada

**Keywords:** Community Wellness Worker, Mental Wellness, Indigenous populations, Métis, Mental Health Service Delivery, Community Health Worker

## Abstract

**Background:**

There is a growing interest in employing community wellness worker models in Indigenous populations to address inequities in healthcare access and outcomes, concerns about shortage in health and mental health human resources, and escalating burden of chronic and complex diseases driving significant increase in health services demand and costs. A thorough review of Indigenous community wellness worker models has yet to be conducted. This rapid review sought to outline the characteristics of a community wellness worker model in Indigenous contexts across the globe, detailing factors shaping implementation challenges and success.

**Methods:**

A rapid review of the international peer-reviewed and grey literature of OVID Medline, Global Index Medicus, Google, and Google Scholar was conducted from January to June 2022 for Indigenous community wellness/mental health worker models and comparative models. Articles were screened and assessed for eligibility. From eligible articles, data pertaining to study design and sample; description of the program, service, or intervention; model development and implementation; terminology used to describe workers; training features; job roles; funding considerations; facilitators and barriers to success; key findings; outcomes measured; and models or frameworks utilized were extracted. Data were synthesized by descriptive and pattern coding.

**Results:**

Twenty academic and eight grey literature articles were examined. Our findings resulted in four overarching and interconnected themes: (1) worker roles and responsibilities; (2) worker training, education, and experience; (3) decolonized approaches; and (4) structural supports.

**Conclusion:**

Community wellness worker models present a promising means to begin to address the disproportionately elevated demand for mental wellness support in Indigenous communities worldwide. This model of care acts as a critical link between Indigenous communities and mainstream health and social service providers and workers fulfill distinctive roles in delivering heightened mental wellness supports to community members by leveraging strong ties to community and knowledge of Indigenous culture. They employ innovative structural solutions to bolster their efficacy and cultivate positive outcomes for service delivery and mental wellness. Barriers to the success of community wellness worker models endure, including power imbalances, lack of role clarity, lack of recognition, mental wellness needs of workers and Indigenous communities, and more.

**Supplementary Information:**

The online version contains supplementary material available at 10.1186/s12939-024-02185-5.

## Background

Mental health disparities between Indigenous and non-Indigenous people in Canada stem from cultural genocide and the continued impact of colonization [1]. These health inequities emerge from deep rooted disparities in the social determinants of health caused by social exclusion, political marginalization, and historical trauma, and are avoidable and unjust [2, 3]. Furthermore, additional factors such as public health and climate crises can exacerbate existing mental health issues, compounding their detrimental effects.

In recent years, many Indigenous populations across the globe have begun implementing community wellness worker (CWW) models as a promising approach to address mental health disparities. This growing interest around CWWs (also known as community health workers or community mental health workers in the literature) is being driven by poor healthcare and mental health service access and rising mental health concerns among Indigenous peoples in Canada. Moreover, concerns about shortage in local health and mental health human resources, and the escalating burden of mental health challenges and chronic and complex diseases that has led to significant increases in health services demand and costs in many developed countries [4]. CWW models offer promising solutions to tackle system barriers that impede the delivery of equitable, safe and appropriate mental health services and programs for Indigenous community members [5]. Noted barriers include high turnover, high rates of burnout, and poor retention of mental health providers and support workers in Indigenous communities, resulting in inconsistent and sporadic delivery of care [5]. Another significant barrier is fear or mistrust of Western systems or mental health professionals felt by Indigenous community members due to prior experiences with racism and discrimination [6–8]. Accessibility and availability of culturally-appropriate and culturally-safe mental health services and supports is also often particularly lacking for Indigenous populations [9]. Moreover, communities often face a lack of health infrastructure, and high costs for care provision [8, 10–18].

There are several concepts and contextual considerations that underpin this review. For example, the concept of mental health promotion, which “involves actions to create living conditions and environments that support mental wellness across the lifespan and allow people to adopt and maintain healthy lifestyles” [19]. Multisectoral action is needed across various environments, including homes, schools, communities, and the justice system. This can be achieved through culturally-safe, strengths-based programs, services, and policies that prioritize the mental health and wellness of children, adolescents, and those at risk. Mental health experiences and strengths vary among Indigenous communities and this variation reflects the distinctiveness of Indigenous peoples’ histories, languages, cultures, environments, and worldviews [20–22]. Thus, alternative service delivery models need to be explored with each respective community to determine local appropriateness and feasibility, while also upholding each Nation’s right to self-determination [20, 21]. Health determinants are also observed across the lifespan and across multiple generations to influence health experiences and outcomes [23]. Indigenous CWWs are well-placed to address system gaps in mental health service delivery because of their insight into the specific cultural needs and protocols of their communities as well as the person-centered needs of individual community members. They have a unique ability to bridge the disconnect with non-Indigenous mental health professionals and facilitate the process of accessing mainstream mental health services by crossing the cultural barriers [24].

Despite the increasing utilization of CWW models, a thorough review of these initiatives has yet to be conducted. There is a notable gap in knowledge as to what constitutes a CWW in Indigenous contexts, what makes them successful, and what barriers or facilitators they may face for implementation. A comprehensive exploration of Indigenous CWW models could be instrumental in informing and supporting development and implementation of a model in mental health service provision with Indigenous communities. Consequently, this review seeks to answer the following research questions:

1. What is known about successful Indigenous community wellness/mental health worker models (e.g., what are the key characteristics that made them successful)?
2. What specific roles are Indigenous community wellness/mental health workers responsible for and what are the training requirements?

## Methods

A rapid review of the international peer-reviewed and grey literature was conducted from January to June 2022. Rapid review methodology was chosen because there was an urgent need for evidence to inform a CWW model to promote mental health among Métis residents living in the Regional Municipality of Wood Buffalo in northern Alberta, Canada. The Métis are an Indigenous people whose historical homelands includes Canada’s three Prairie Provinces, as well as parts of British Columbia, the Northwest Territories, and Northwest Ontario. They have a shared history and culture, deriving from specific mixed European (primarily French, Scottish, and English) and Indigenous ancestry.

In the Summer of 2019, a two-day Indigenous Mental Health Forum in the Regional Municipality of Wood Buffalo was hosted by McMurray Métis Local and University of Alberta’s School of Public Health through a community-academic partnership [25]. A key outcome from the forum was direction to design a holistic and culturally-rooted mental health system in the region. A Métis Mental Health Advisory Committee was formed in 2022 to guide the overall development and implementation of a Métis mental health action plan in Wood Buffalo. The establishment of a holistic service delivery model focused on community wellness, that is designed and delivered by Indigenous peoples and prioritizes training of local Indigenous health workers, was one of the priorities identified in the plan. Hence, this review was conducted in efforts to build an evidence-informed CWW program for Métis residents in the region. The advisory committee provided guidance on the review question and interpretation and contextualization of the review findings. Additionally, a Métis Elder, who is also employed as a program manager (BF) at McMurray Métis Local and co-author on this article, was a part of the academic rapid review process and met bi-weekly with the research team while working on this research initative. Moreover, the lead author (KF) has Indigenous ancestry and is from the Regional Municipality of Wood Buffalo, and the principal investigator (SM) has supported community-based research in the region for 8 years and has a long-standing relationship with McMurray Métis Local and partnerships with other communities. Integrating Indigenous worldviews and ensuring Indigenous engagement and leadership in this work was an important step in our approach to conducting this review.

We utilized the rapid review methodology outlined by the National Collaborating Centre for Methods and Tools [26]. An expert health sciences librarian (SC) conducted peer-reviewed literature searches in OVID Medline and Global Index Medicus using specified search terms detailed in Appendix A. The term ‘community mental health worker’ was chosen given broad usage in the literature. We excluded terms which can be classified as ‘health professional’ as defined by the WHO’s mapping of occupations [19]. The terminology used in each article is highlighted in the results section under Table 1.

**Table 1:** Terminology used to refer to CWWs in the literature

Terminology for CWWs	Number of Articles	Source(s)
“Aboriginal/Indigenous mental health workers”	*n* = 11	[7, 13, 30–34]
“Community health agents” or “Community health worker”	*n* = 2	[35, 36]
“Child and adolescent mental health workers” or “ACCESS OM Youth Workers (AYW)”	*n* = 2	[10, 37]
“Aboriginal health workers” or “Aboriginal health and welfare workers” or “Aboriginal and Torres Strait Islander health workers”	*n* = 2	[24, 38]
“Aboriginal Mental Health Liaison Officer”	*n* = 1	[8]
“Community Health Aide” or “Behavioral Health Aide”	*n* = 1	[11]
“Personal helpers and mentors”	*n* = 1	[39]

The Population, Intervention, Comparator, and Design (PICOD) framework guided our inclusion and exclusions criteria (Table 2). Eligible peer-reviewed literature included systematic reviews, literature reviews, primary studies, dissertations, and theses published in English, without date or geographical exclusions. Articles for inclusion comprised those that: [1] focused on Indigenous peoples; [2] were set within institutional or community-based settings; [3] showcased outcomes directly related to care delivered by Indigenous community wellness/mental health workers and similar roles; [4] outlined the components of Indigenous community wellness/mental health worker models; [5] discussed the roles and responsibilities of community wellness/mental health workers; and [6] touched on economic impacts and funding allocation.

**Table 2:** PICOD framework

Population	Indigenous peoples globally
Intervention	Focused on Indigenous community wellness worker (and alternative terms for ‘community wellness worker’ outlined in Table 2) programs and services
Comparison	N/A
Outcome	Improvements in Indigenous mental health; Reducing barriers and stigma related to seeking mental health care; Increase availability of services and culturally safe care (reach); Roles of community wellness worker (e.g., patient navigation, case management, cultural appropriate education, help tap into community and cultural assets)
Study Design	Empirical studies, empirical review studies, theoretical studies, implementation studies

**Fig. 1:**
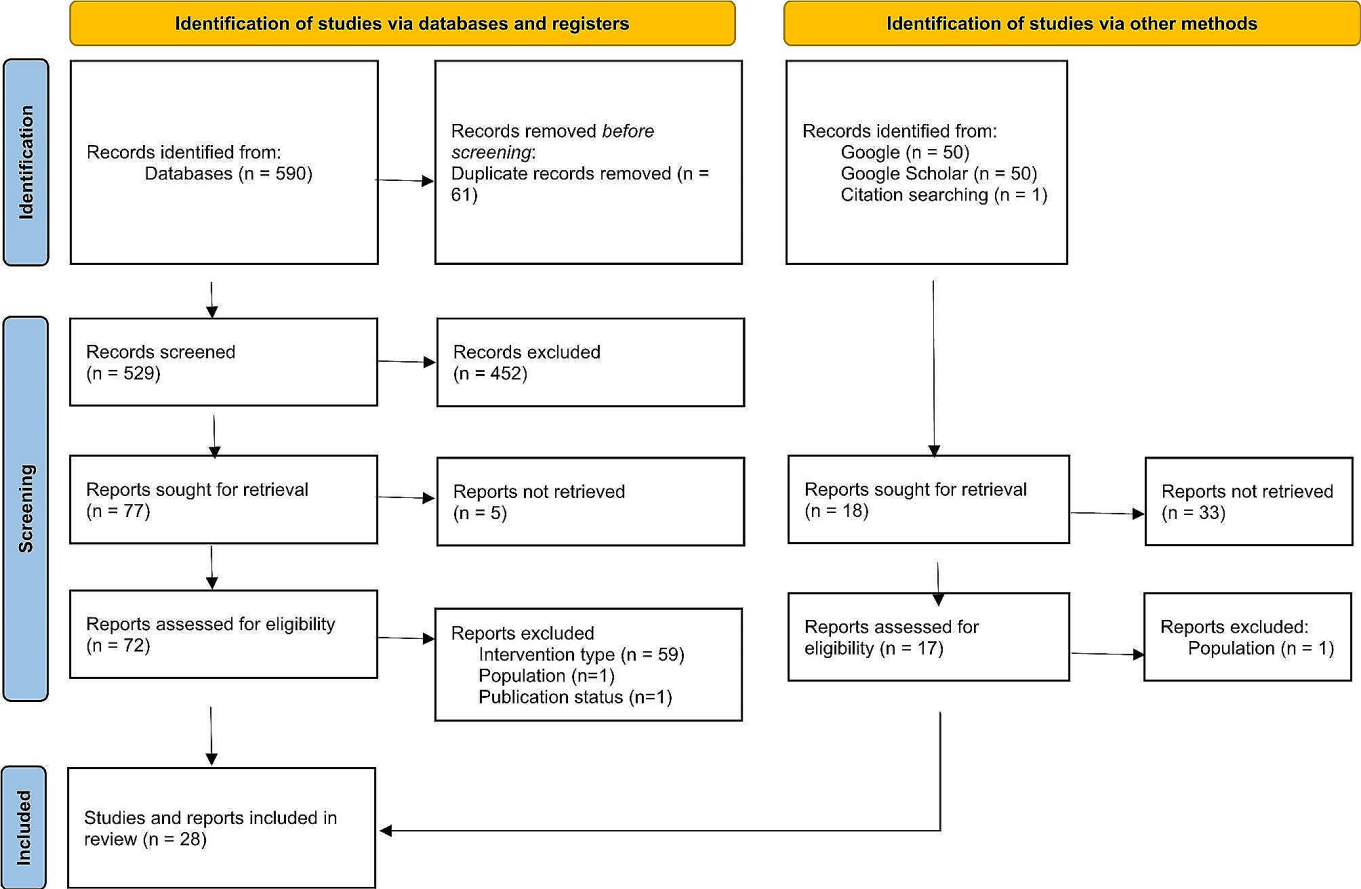
PRISMA flow diagram Page MJ, McKenzie JE, Bossuyt PM, Boutron I, Hoffmann TC, Mulrow CD, et al. The PRISMA 2020 statement: an updated guideline for reporting systematic reviews. BMJ 2021;372:n71. 10.1136/bmj.n71. For more information, visit: http://www.prisma-statement.org/

Title and abstract screening were done by authors (GP, HR), full text screening by authors (GP, KF). Any disagreements regarding article inclusion were resolved by discussion and with senior researchers (KF, SM) until a consensus was reached. See Fig. 1 for the PRISMA diagram and Additional file 1 for the PRISMA-S checklist [see Additional File 1]. Grey literature sources were identified via Google and Google Scholar; results were screened utilizing the same inclusion criteria applied to the peer-reviewed literature. Moreover, we searched for “Indigenous Community Mental Health” job postings to supplement the discussion around CWW models. Data extraction used Microsoft Excel, covering study design; program/service/intervention descriptions; terminology for Indigenous community mental wellness workers; training features; job roles; funding considerations; facilitators and barriers to success; key findings and outcomes; and any information on models utilized. Additional files 2 and 3 detail the academic literature and grey literature search strategies respectively [see Additional Files 2 and 3].

Study quality was assessed by one author (ES) using Critical Appraisal Skills Program (CASP) quality assessment tools [27], to evaluate research clarity, methodology, alignment of findings with research objectives, and local applicability [27]. Data synthesis followed Miles and Huberman’s [28] qualitative thematic analysis, with open coding by authors (KF, GP, HR) and verification by the senior author (SM) and a community partner (BF). Our thematic analysis was further informed by principles in grounded theory [29].

## Results

A total of 28 studies and reports were selected for inclusion in this review, with 11 from databases and registers and 17 from other sources. Figure 1 presents the PRISMA flow of articles selected through the review. The search obtained a total of 590 peer-reviewed records, which were reduced to 529 after removing duplicates. Following title and abstract screening, 77 records moved forward to the full-text review stage, and of these, 11 were included in the review. The grey literature search generated 8 sources consisting of reports or website content and an additional 8 peer-reviewed studies, while one other relevant study was identified through a citation search. The characteristics of the articles along with the themes identified through analysis are summarized below.

### Article characteristics

Majority of this literature was published after 2010 (*n* = 17) and involved Aboriginal and Torres Strait Islander peoples (*n* = 23), Indigenous peoples in Canada (*n* = 3), Indigenous Brazilians (*n* = 2), and American Indians/Native Americans (*n* = 1). The sample populations reported by studies included Indigenous youth, adults, general community members, Indigenous community wellness workers (CWWs) themselves, and/or program clients and caregivers. Most articles (*n* = 12) presented evaluation outcomes of Indigenous CWW models, employing quantitative (*n* = 4), qualitative (*n* = 3), and mixed-methods (*n* = 4) approaches. Six articles were purely descriptive, detailing CWW models, including model content and processes of development and implementation. Two articles concerned implementation science methods to study strategies that facilitate the uptake and sustainability of CWW programs. Additional, non-refereed literature (*n* = 8) consisted of non-peer reviewed program evaluation (*n* = 3), research (*n* = 1), and implementation reports (*n* = 1), along with website content (*n* = 2) and a descriptive report (*n* = 1). Additional file 4 includes Tables 3 and 4 that provide and overview of the peer-reviewed studies and grey literature respectively [see Additional File 4].

### Themes

**Fig. 2:**
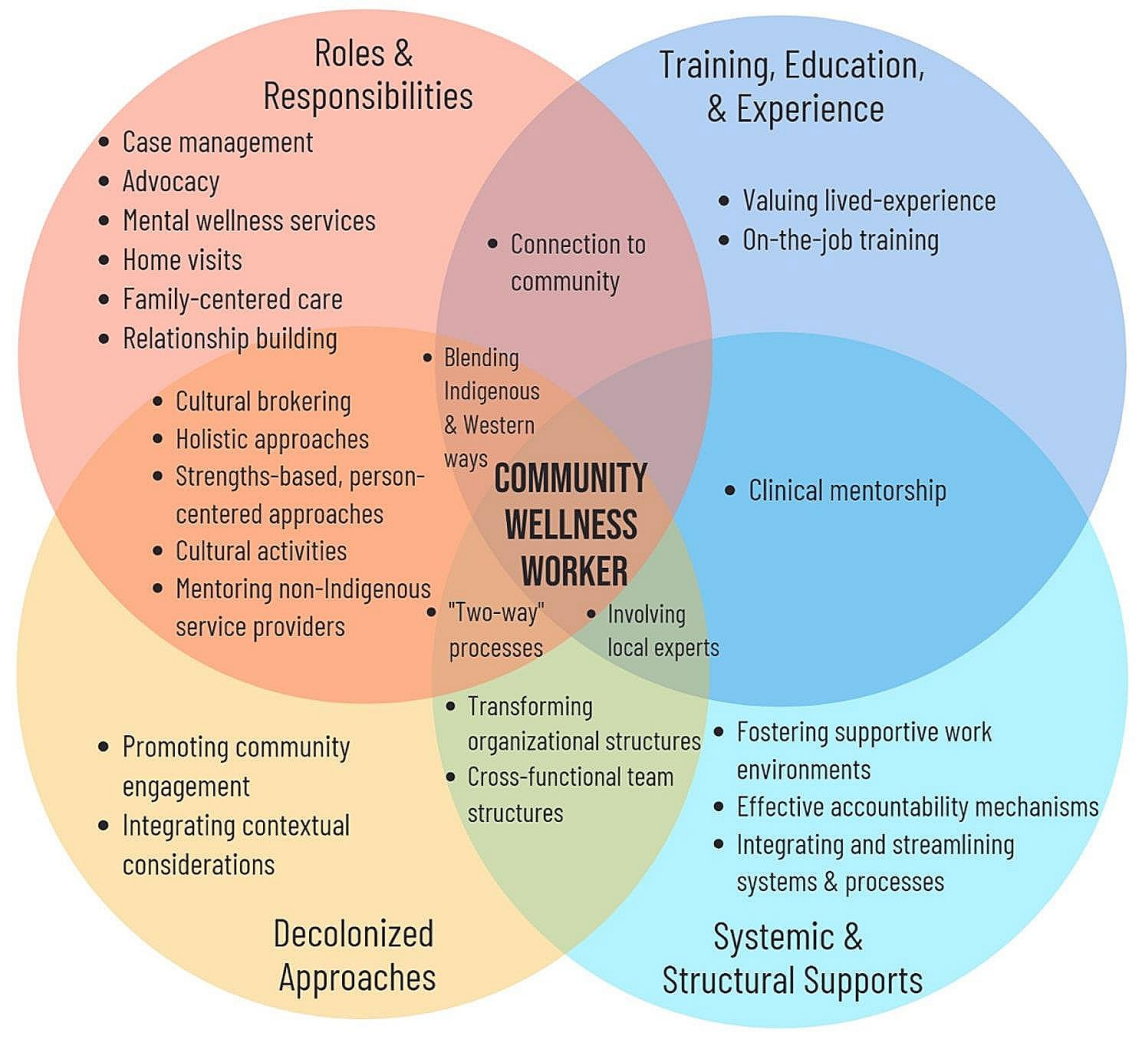
Emerging themes and sub themes to inform the Indigenous community wellness worker

The findings from the academic and grey literature sources are presented as four overarching and interconnected thematic areas. These thematic areas focus on the following dimensions of community wellness/mental health worker models: [1] worker roles and responsibilities; [2] worker training, education, and experience; [3] decolonized approaches; and [4] structural supports. In this section we describe these themes, highlight components of CWW models that exemplify each theme, and discuss the enablers and barriers to CWW model success as they relate to each theme. Figure 2 further details the themes and subthemes that contribute to the CWW model, and interrelationships among themes.

### Worker roles and responsibilities

In the literature, terminology used to refer to community wellness/mental health workers were diverse. Terminology was typically reflective of CWW roles and responsibilities (i.e., “Behavioral Health Aide” or “Aboriginal mental health worker”), described target populations (i.e., “Child and adolescent mental health workers”), and/or gave insight into how they operate alongside mainstream mental health services (e.g., “Aboriginal Mental Health Liaison Officer”). One author noted that the terminology used in their CWW model “[pertained] to specific funded programs and/or use the same terminology used by published literature when providing existing examples” [11]. Most CWW models referred to workers as “Aboriginal/Indigenous mental health workers” (*n* = 11). Terminology for CWWs is further detailed in Table 1.

Descriptions of the roles and responsibilities of CWWs featured prominently in articles and reports examined for this rapid review. The identified roles and responsibilities of CWWs are diverse, blending conventional health and social service worker functions such as case management, advocacy, and mental health intervention supports with other functions beyond the conventional, such as relationship building, a focus on holism and fostering strengths, providing both person- and family-centered care, and Indigenous wellness approaches involving ceremony, land-based activities, incorporating art, and more. They are frequently involved in a variety of community-based programs. Notably, the position of a CWW requires considerable flexibility and adaptability to be able to meet the diverse, complex, and high needs of Indigenous communities, especially where access to other supports or services is limited or absent. They frequently act as advocates and as agents of social change. CWWs have strong roots in the community they serve and utilize local knowledge in available mental health, social, and cultural resources in their efforts to support the wellness of community members.

CWW roles and responsibilities cross the full spectrum of case management services, including involvement in intake processes, conducting assessments, case documentation, service planning and coordination, monitoring and evaluation, navigation across services, client advocacy, supporting transitions, conducting follow-up, and more [10, 30, 35, 40, 41]. CWW models are commonly structured so that workers carry out case management roles alongside clinicians such as nurses, mental health therapists, and/or physicians who are typically non-Indigenous and situated outside the community.

Articles noted how CWWs bring additional capacities to supplement conventional case management. Namely, CWWs “act as a cultural link between patient, family and service providers” [30] helping to facilitate communication and understanding between service providers and community members [24, 33, 36]. CWWs aid in the cultural adaptation of mental wellness tools, resources, and intervention strategies and also help mainstream service providers adapt their ways of working to better meet the needs of communities [11, 24, 30, 34, 36, 37, 42, 43].

CWWs are important gatekeepers who are well-situated within the community, both physically and relationally, to reach out, identify those in need of support, and connect them to needed services or supports [32, 37]. In this sense, CWWs facilitate entry into provider settings, a role that has been described as a “bridge” between the community and care providers [8, 35]. CWWs also vet and assess new service providers and will “vouch” for outside professionals that they deem to be “okay”, facilitating community member access to professionals who are culturally safe [33].

Articles underscored how the unique advocacy role of CWWs was closely connected to their position as a “cultural broker” between community and service provider [8, 11, 40, 44]. For example, as cultural brokers, CWWs would “[reflect] Aboriginal patients’ concerns as part of their own concerns, giving voice to patients who may feel reticent to access the service and speaking up with confidence when government health services were taking an approach that’s not ‘our way’.” [24]. They also undertake activities to mentor non-Indigenous mental health service providers to improve cultural awareness and safety [7, 8, 10, 14, 24, 30, 32–34, 40, 41]. For instance, Robinson and Harris [40] note how CWWs “have significantly contributed to other practitioners’ ability to understand background issues and cultural themes relating to clients’ problems.” (p. 52).

CWWs are key advocates for system-level change to better meet the needs of community members. Notable changes facilitated by CWWs include cultural adaptions to mental health interventions [10, 31, 32, 42]; integration of dominant-culture knowledge and therapeutic approaches with local cultural knowledge [14]; introduction of new interventions and supports, especially those employing Indigenous approaches to wellness [30, 37]; changes to training initiatives [10, 11]; and changes to team structures and workforce planning [14].

CWWs are often actively involved in direct mental wellness service provision, delivering a diverse array of interventions to community members to promote mental wellness. Interventions conducted by CWWs include those recognized within Western, biomedical approaches such as interventions preventing suicide and self-harm, mental health first aid, substance use support, crisis intervention, family counselling, psychotherapy (individually or in conjunction with a clinician), and conflict resolution [7, 34, 40]. Articles noted how CWWs also facilitated other types of mental wellness interventions beyond what is typically provided by existing mental health services. These interventions involved the integration of Indigenous cultural, spiritual, traditional healing practices through, for example, art, land-based activities, spiritual understandings, humour as medicine, movement, and the inclusion of Elders [11, 14, 31, 32, 34, 37, 38, 42, 43]. Another notable mental wellness support involved home visits and the involvement of family [11, 14, 15, 35, 36, 40, 42, 44].

The concept of holism permeated all aspects of the roles and responsibilities of CWWs in the articles reviewed. Overall, CWWs have an important role in promoting holistic approaches to mental wellness, acknowledging the importance of balance between the mind, body, spirit, and emotions while providing care to the whole person [14, 31, 36, 43]. As noted above, CWW models aim to promote mental wellness of the individual in while also considering relationships with people and environments that surround them, especially family, household, and wider community contexts [14]. For instance, the advocacy role of a CWW did not exist solely within the silo of mental health services but extended to other aspects of clients’ lives to include liaising with the justice system and assisting with meeting clients’ basic needs for food, clothing, transportation, and more [7, 40]. Authors also noted that holistic models must consider the impacts of experiences of trauma and loss linked to colonization [31, 38]. Holism was supported by integrated service delivery models, where general health care providers, mental health service providers, and Indigenous wellness supports collaborate and work together to be able to respond to the needs of the whole person [42].

Furthermore, CWW roles and responsibilities can also encompass more than just a focus on service delivery and outcomes. They also prioritize the thoughtful organization of programs, training initiatives, self-care strategies, and other elements to ensure that the needs of both community members *and* workers are met effectively [14, 35, 38, 43]. This subtheme of supportive work environments is addressed further in a later theme.

Authors noted several important elements linked to the roles and responsibilities of CWW that acted to facilitate or impede the success of CWW models. Home visits and involvement of the family in the provision of mental wellness supports were highlighted as important facilitators for success. Authors noted how home visits facilitate access to needed supports by circumventing important barriers to care like wait times, lack of transportation, or unwillingness to visit hospitals or clinics. Moreover, home visits encourage relationship building and enable the extension of individual-level interventions to the family context. As noted by Waidman, Costa, and da Paiano [36], a home visit “makes it possible to understand the family dynamics and to check the possibility of involving the family in the treatment offered to the service user” (p. 1173). It was noted that home visits enable integration of intervention between client, their families, the community, and health care professionals in the area [36].

The partnership between clinicians and CWWs was noted as an important facilitator of success because knowledge and expertise can be shared, more clients can be reached, clinicians can be relied upon for back-up in dealing with mental health problems that are beyond CWW capacity, and CWWs are able to build confidence and knowledge in the assessment and treatment of mental illness [7, 10, 37, 43]. Conversely, inequality between CWWs and clinicians was a notable barrier, which was often experienced as a lack of recognition for their work, skills, and knowledge; experiences of stigma, racism and discrimination; inequitable status and remuneration; and more [12, 24, 31, 32]. CWWs reported that these inequities hampered collaboration, contributed to poor work conditions, and exacerbated job dissatisfaction.

The relationships that CWWs establish and maintain with wider communities was noted as an important facilitator to success. This relationship that CWWs have with community is often pre-existing, as many are members of or have close ties to the communities in which they work [8, 11, 12, 15, 35, 36, 39]. McKenna et al. [8] note that the ability to “broker such engagement required an in-depth knowledge of referral opportunity in the community” (p. 5). CWWs’ close ties with community also enables them to anticipate and mitigate potential challenges including concerns around privacy, confidentiality, stigma, and local strengths, resilience, and risks to mental wellness [8, 11, 39]. Lastly, CWWs’ shared cultural background comes with better cultural awareness and safety [36, 39].

On the contrary, close ties to community can also present a number of barriers to CWW success. CWWs reported challenges separating their professional and personal lives, which can take an emotional toll and contribute to burnout [8, 10–13]. Strong family and kinship relationships between CWWs and clients may introduce complexity to who can appropriately provide care [11, 24, 39]. As noted by de Souza et al. [35], the strong bonds CWWs have with community “put them in a good position of reference, however, did not allow the detachment necessary to maintain their privacy” (p. 209). The small size and close-knit nature of communities may also make it difficult for CWWs to maintain client privacy [37].

Authors noted that the unique roles of CWWs provided a number of benefits in the provision of clinical mental health services in Indigenous communities, including early case detection, expansion of access, higher rates of engagement in services, improved patient adherence to treatment, enhanced efficacy of services, more thorough case documentation; reduced loss to follow-up, improved relationships and trust between clients and service providers, and better management [30, 33, 35, 40, 43].

Insufficient role clarity emerged as a significant obstacle, leading to job confusion, unclear expectations, ineffective collaboration between different professions, subjecting CWWs to criticism from both clinicians and community members. This situation often leads to CWWs experiencing excessive workloads or being underutilized, ultimately contributing to burnout and high turnover rates [7, 12, 24, 36, 38]. Several articles noted that unclear CWW roles and responsibilities put them at risk of becoming “tokenistic” and being relegated to what mainstream service staff deemed as Indigenous or cultural issues only, instead of embracing their full, diverse skillset [7, 12]. This lack of role clarity can also create barriers to the expansion of CWW models into other communities [7].

### Worker training, education, and experience

The literature examined highlighted worker training, education, and experience features within CWW models. As noted above, CWW models and their respective worker training, education, and experience features are diverse; however, they are connected by several shared attributes. Namely, CWW models place a high value on the lived-experience of workers over formal education, focus heavily on on-the-job training, and strive to integrate Western, biomedical training/education methods with Indigenous approaches.

While the term CWW has the potential to encompass workers with diverse formal qualifications such as nurses, social workers, or psychologists [31], most CWWs do not possess formal degrees or other formal pre-service training from recognized academic institutions [33, 45]. Notwithstanding, CWW training includes investment in on-the-job training [10]. CWW models place value on workers’ lived experiences, their collective cultural and linguistic knowledge, insights into community resources, and their roles as trusted and respected helpers [37]. Lived-experience was highlighted as critical asset that CWWs bring to mental wellness services in their communities. CWW models purposefully recruit workers who are from community or have significant familiarity with the community, specifically because their lived-experience and relationships within communities are pre-requisites for their important roles and responsibilities as trusted cultural brokers between community and mainstream service providers [8, 11, 40, 44]. Furthermore, CWWs possess in-depth knowledge of community dynamics, kinship patterns, cultural values, attitudes, mental wellness challenges and resiliencies, language, and client expectations that conventional service providers often do not [11]. CWWs are uniquely able to “tap local cultural assets and resources to promote mental wellness” [11].

On-the-job training was highlighted as critical to supporting CWW model success. It was noted that many CWWs have limited prior access to professional development and training. Requiring pre-service qualifications can thus limit the talent pool and adversely impact the ability to recruit CWWs [14]. This is especially pertinent in contexts where recruitment and retention of Indigenous staff, especially recruitment in remote communities or of younger workers, is already a significant challenge [14, 15]. Scholars highlighted how this approach can aid in fostering local capacity, enhance their career opportunities, and increase access to higher incomes for CWWs [14, 15, 39, 45, 46]. It is also important in planning for the future of Indigenous mental health services [14]. Moreover, scholars noted that the on-the-job training approach offered full-time employment and support to gain a degree which attracted candidates to the position and increased job competence [15]. Ongoing access to a wide-range of training opportunities to meet the complex, diverse, and ever-evolving mental wellness needs in community was also a noted facilitator for success of CWW models [35]. Uptake of on-the-job training initiatives was linked to CWWs’ “high levels of commitment to self-development and assisting the Aboriginal community” [14].

On-the-job training and education also builds worker confidence and knowledge related to Indigenous mental health and in addition acquire practical skills directly relevant to their work [14]. On-the-job training often includes those recognized within Western institutions along with culturally-based methods. Similarly, it commonly involves structured experiential learning through clinical placements or clinical mentorship [10]. On-the-job training for CWWs often involve opportunities to receive certificates, degrees, or professional designation [13, 15, 24, 30, 31, 38, 39]. On-the-job training and education for CWWs also involved structured (e.g., clinical observation/supervision, case reviews, practicums) and unstructured (e.g., ability to consult clinicians as needed) mentorship from clinicians [7, 10, 24, 34, 36, 39].

Clinical mentorship of CWWs was a noted enabler of success. The literature frequently cited how CWWs highly valued the clinical support provided by nurses, physicians, psychiatrists, and therapists, especially because exposure to clinical training and education opportunities might otherwise be challenging to access [10]. Clinical mentorship supported CWWs in attending to mental health concerns of community members that they may have otherwise feared or felt unqualified to handle [32, 34, 36]. It also created structural benefits to CWW models, facilitating improved organization of local programs by encouraging monthly scheduling, clinical and professional goal setting, and creating regular processes for program planning and problem-solving [10, 39]. Scholars did note, however, that the success of clinical mentorship is dependent on the establishment of good, trusting, and accessible relationships between clinicians and CWWs [7, 10]. Reported barriers to the success of clinical mentorship included inequitable relationships, role confusion, heavy workloads, high staff turnover for both CWWs and clinicians, and incongruent views about local mental health needs and how to address those needs [7, 8, 10, 32].

Articles highlighted several critical factors associated with CWW training, education, and experience that either supported or hindered their success. Connection with, and responsibility to, community was reflected by many articles as essential to developing trust with clients and enabling CWWs to be effective in their work [11, 24, 33]. Building rapport with clients through knowledge of the community and recognising family connections was noted by scholars to enable the role of CWWs [24, 33]. On the other hand, being members of a community with a disproportionate burden of mental wellness challenges was noted as another possible barrier. Some scholars highlighted that CWWs had a high likelihood of possessing personal histories of trauma, violence, and abuse along with collective experiences of trauma as Indigenous peoples, which may put them at risk of health and mental wellness challenges that could impact their work and learning experiences [38]. Of particular note was how legacies of prior educational experiences could negatively impact experiences with on-the-job training [38].

Lastly, adapting and tailoring training initiatives to be culturally sensitive and responsive to the needs of CWWs, their clients and community was also expressed as an important facilitator to success [42, 43]. Scholars reported that integration of culturally-specific information, tools, strategies, and resources into training improved communication and understanding between CWWs and their clients, helped to “demystify and simplify the management of mental health and well-being concerns of Indigenous…clients” [43]. The ways in which training initiatives were adapted included: involvement of Indigenous co-facilitators [43, 47], a focus on strengths [42], flexible training formats [14, 40, 43], introducing Indigenous ways of learning [38, 42], including Indigenous concepts of mental health and well-being [31, 42, 43], and incorporating sociopolitical history of Indigenous peoples [15, 38, 40]. Flexible accreditation processes for on-the-job training opportunities for CWWs was another important facilitator [11].

### Decolonized approaches

Decolonized approaches in CWW models were a prominent theme in the literature. Decolonized approaches involve centering Indigenous knowledge, cultural views, values, and practices into mental health services while also challenging and resisting dominant, colonial approaches, structures, and power dynamics [14, 48]. In the context of health systems, decolonization prioritizes Indigenous-led initiatives that confront enduring colonial legacies. This may involve initiatives such as cultural humility training, which views safety as an outcome of decolonization, along with anti-racism efforts, recognizing power imbalances, addressing structural violence, and committing to truth and reconciliation [49]. While few authors explicitly identified employment of decolonized practices into CWW models, such practices were nonetheless evident across all articles analyzed in this review. The literature highlighted decolonized approaches within CWW models that prioritized strong Indigenous involvement and community engagement, the blending of Indigenous values and wellness perspectives with Western practices, integrating and prioritizing contextual considerations, and the transformation of organizational structures to align with Indigenous principles.

Most articles mentioned community engagement as important to the development and implementation of CWW models. We categorized community engagement as supportive of decolonization because some of the stated goals of community engagement processes utilized in CWW models included improving service relevance, appropriateness, effectiveness, and uptake; to uphold Indigenous self-determination; and to integrate Indigenous values, knowledge and approaches into wellness promotion [8, 11, 24, 30, 42]. This approach supports the adage “nothing about us, without us” that many Indigenous leaders and communities emphasize when it comes to research, program development, and policy for Indigenous peoples [50, 51]. The establishment of a local workforce consisting of Indigenous CWWs inherently advances community engagement in mental wellness services. As highlighted by O’Keefe et al. [11], CWWs serve as built-in advocates, promoting the specific needs and priorities of their respective communities. Engagement was commonly achieved by consulting with CWWs themselves—as knowledgeable members of the community with insight into community needs—about program design and implementation [13, 24, 32, 34, 47]. More rarely did engagement extend to consulting with clients, their families, or the wider community [10, 30, 37, 38, 42, 43]. Engagement at times involved consultation with both Indigenous stakeholders (such as clients, CWWs, and community leadership) *and* non-Indigenous stakeholders (including mainstream health service providers, clinicians, and government officials) [10, 34, 42]. Notably, none of the initiatives reviewed appeared to be Indigenous-led, rather they incorporated Indigenous peoples and organizations and centred their work around Indigenous concepts. However, few articles explicitly described their engagement process, rendering it difficult to know the extent and quality of engagement with Indigenous peoples and leadership in the development of some CWW models reviewed in the literature.

Blending Indigenous ways of knowing, being, and healing with Western, biomedical approaches was another decolonial approach commonly employed in CWW models. This blending has been touched upon in many of the themes already discussed above. It was highlighted in the roles and responsibilities of CWWs who work alongside clinicians to provide conventional mental health services while also incorporating Indigenous cultural, spiritual, traditional healing practices [11, 14, 31, 32, 34, 37, 38, 42, 43]. The training, education, and experience valued within CWW models prioritizes workers’ shared cultural knowledge, connection to community, and lived-experiences while also aiming to equip them with clinical knowledge and skills [10, 11, 24, 33]. Scholars argued that it is necessary to “[blend] Indigenous concepts of health and well-being with non-Indigenous ways of understanding and treating illness in order to develop services which are appropriate to Indigenous peoples” [43].

Integrating contextual considerations into CWW models was also expressed as an important decolonial component. Contextual considerations included gaining the knowledge of a community’s unique risks and protective factors; ensuring that the needs of workers are addressed; respect for and adherence to local protocol; involvement of local experts/Elders; developing culture-centered understanding of mental wellness and healing, building and maintaining relationships, contemplating historical impacts; and considering trauma, unresolved grief, and physical health needs [11, 14, 15, 24, 33, 34, 37, 40, 46]. For instance, in an evaluation of a CWW pilot in Australia, authors highlight the need to consider how local health needs may impact mental wellness supports offered by CWWs and how these local issues can also impact workers:

“The high degree of physical ill health had a major impact on the mental health of this population. Physical health problems added further stress to patients’ and families’ lives and were potential complicating factors when diagnosing and treating psychiatric conditions…. Aboriginal staff who worked at Durri also struggled with their health, which meant that they would receive medical assistance, including psychiatric care, at their own workplace.” [33].

Other scholars note that CWW models must incorporate means to address the historical and contemporary contexts of being Indigenous. Lauw et al. [38], for example, highlight that programs must not assume all CWWs are aware of the impacts of historical trauma resulting from colonization. Mechanisms to address this history in training and in the workplace can re-frame for CWWs what it means to be Indigenous, shift away from a sense of shame, and prevent CWWs from becoming triggered during work or training [34, 38]. Such mechanisms may include regular debriefing, workshops, peer support, and tapping into cultural approaches to relieve stress [14, 34, 35, 38, 43].

Another important theme with respect to decolonizing approaches is a focus on strengths-based and person-centered approaches [14, 35, 39, 42, 43]. Commonly Indigenous communities have been looked at from a deficit lens, which has resulted in paternalistic western health programming and perpetuation of negative stereotypes. Articles noted how CWWs employed methods that built upon and fostered the existing strengths and capacities of the community members they serve. For example, a CWW model designed to deliver supports to community members around substance use applied a strengths-based care planning process outlined in a “mental health stay strong care plan package” [43]. Other strengths-based approaches included involving important and supportive family members, reviewing client strengths utilizing pictorial tools, providing motivational counselling, and collaborative goal setting [35, 39, 42].

Decolonized approaches within CWW models also involved the transformation of organizational structures to align with Indigenous principles. CWW models paid special attention to power relations and workplace hierarchies. As noted already above, many scholars underlined the importance of promoting equality between clinicians and CWWs [12, 24, 31, 32]. CWW models aimed to stray away from workplace hierarchical structures typical within Western customs. Instead, they fostered collective decision-making, consensus-building processes, and cooperation between all stakeholders. For example, several articles noted “two-way” processes within CWW models that emphasize the reciprocal, supportive, and mutually beneficial partnership between CWWs and mainstream mental health service providers [34]. These “two-way” processes promote more equitable distribution of power and decision-making, where both parties have a voice and influence in shaping the partnership and its outcomes [8, 24, 40, 47]. This “two-way” perspective extends to accountability relationships, where CWW models emphasize a multidirectional flow of accountability, and not merely a ‘downwards’ flow from funders, planners and providers towards workers, service users, or communities [24].

Cross-functional team structures encompassed another decolonized organizational structure employed within CWW models. Cross-functional team structures supported task-shifting, cross-training, interdisciplinary/multidisciplinary teams, and overall team integration. Task-shifting is a practice in which certain tasks are delegated to individuals who have undergone shorter training periods and possess fewer qualifications. For example, “paraprofessionals or local lay providers may be trained in evidence-based mental health practices…and provide mental health interventions in community settings” [11]. Many articles underlined the value of interdisciplinary/multidisciplinary teams. However, CWW models uniquely create space for team members to transcend rigid professional boundaries and instead embrace a more fluid and collaborative approach that fully utilizes the diverse gifts and capacities of each team member, especially CWWs [24]. CWW models analyzed for this rapid review commonly employed cross-training; however, this primarily involved the exchange of knowledge and skills between clinicians and CWWs [7, 8, 10, 14, 24, 30, 32–34, 36, 41]. In several articles, authors noted how CWWs called for more opportunities to learn from each other as well as Indigenous Knowledge Keepers [32]. Scholars underscored how cross-functional team initiatives prevent burnout; maximize the scope of practice of CWWs; improve CWW versatility, adaptability, team collaboration and peer-based support; and support the continuity of care for clients [11, 14, 37]. Such initiatives were also noted to be vital to ensure that knowledge is passed from senior, more highly experienced workers to younger workers [14]. As noted, scholars caution that fluidity within CWW teams must be balanced with some degree of role clarity and opportunities for recognized career advancement [7, 12, 24, 36, 38].

### Systemic and structural supports

Lastly, discussions of systemic and structural supports within CWW models featured prominently in the literature. Noted structural supports included promoting supportive work environments for CWWs and efforts to integrate and streamline systems and processes within the systems that CWWs operate. As mentioned earlier, CWW models recognized the significance of prioritizing the well-being of workers alongside enhancing their outputs (i.e., productivity, performance, and client/patient outcomes). To establish supportive work environments, emphasis was placed on integrating Indigenous approaches to promote the wellness of workers. These approaches encompassed various initiatives, such as providing opportunities for participation in sharing circles, ceremonies, cultural retreats, and land-based healing [11]. Additionally, the provision of protected and paid time off is implemented to ensure workers’ well-being and cultural engagement [40]. Many articles underscored the importance of ensuring work/life balance of CWWs, along with processes for team building, self-care, debriefing, mentorship, and peer support [14, 34, 35, 38, 43]. Supportive work environments for CWWs are especially important because, as members of the communities they serve, they face the same heightened risk for mental wellness challenges [33, 38]. Moreover, they face additional challenges from having to walk in two worlds [24].

Another dimension of supportive work environments concerned effective mechanisms of accountability. Such accountability mechanisms include those that keep CWWs accountable for the supports they provide to clients by, for example, regular reports, performance evaluations, or case reviews [24, 40]. They also include processes to keep mainstream service providers accountable to communities by data collection and program evaluation; efforts for consistent and meaningful community engagement; mechanisms to report and address experiences of racism; and/or dispute mechanisms to resolve, for instance, unilateral funding or policy decisions [8, 11, 24, 30, 33, 42, 47]. Efforts to integrate and streamline systems and processes within the arenas that CWWs work were also noted to be crucial. In fact, the low level of both vertical and horizontal integration between existing mental health and related services were cited as key drivers for the implementation of CWW models [7]. A key role of CWWs is to act as “cultural interlocutors between clients and service providers….to forge stronger links between the Indigenous community and the various predominantly non-Indigenous providers, coordinating the efforts of other stakeholders toward the program goal of achieving shared primary mental health care.” [7].

Scholars identified several mechanisms to support system integration within CWW models. First, “two-way” processes foster integration via bidirectional learning, support, advisory, mentorship, or supervision between CWWs and clinicians or other mainstream service providers [8, 24, 34, 40, 47]. Multidisciplinary teams support integration through enhanced communication and information sharing, improved timely referral and transition supports, collaborative decision-making, and care coordination [8, 10, 12, 14, 24, 31, 32, 35]. Moreover, designating CWWs as clinical case managers who walk with clients as they navigate through health and social services was reported as an important mechanism for integration of services and improving the continuity of care [32]. Other mechanisms noted by scholars included practices such as case conferencing, buy-in from mainstream service providers, and other practices that build the role of CWWs into the operations of mainstream health and social services [7, 32].

Nonetheless, scholars did note that efforts to integrate services and blend Indigenous with Western approaches presented challenges for CWWs. Multiple papers highlighted how CWWs felt tension between the cultural and community obligations and clinical and administrative aspects of their role. CWWs felt like they were straddling different cultures, which was exacerbated by CWWs’ sense of co-ownership of health because clients are ‘our people’ as well as stringent expectations by Western systems for objectivity and maintenance of professional boundaries [24].

Lastly, another notable structural support was ensuring flexible accreditation processes for CWWs and the training they receive [11]. Such processes support career advancement of CWWs and furthers the recognition of their role and expertise among their mainstream colleagues. Moreover, continuing and dependable funding models that adjust to meet the growing needs of Indigenous populations were also noted to be paramount [11].

## Discussion

This rapid review set out to explore Indigenous CWW models, the characteristics that make them successful, and the roles, responsibilities, and training requirements typically sought from CWWs. Most articles we examined involved CWW models employed in Indigenous contexts in Australia (*n* = 23). One potential reason for this is that in Australia, a longstanding Aboriginal-led movement, rooted in addressing racism within the healthcare system, has actively fought for the incorporation of CWW initiatives into primary health care services across the country since the 1970s and 80s. This development coincided with the establishment of Aboriginal-controlled health services that strongly advocated for the inclusion and recognition of CWWs in the healthcare system [52]. Moreover, as the CWW role developed, emphasis was increasingly placed on their ‘cultural brokerage’ role and on the provision of culturally safe and comprehensive health services to Aboriginal and Torres Strait Islander people [53, 54]. In contrast, with the exception of the Community Health Agents Program (CHAP) program in Brazil, CWW models in other jurisdictions are restricted to small, local programs or pilot projects and have not been applied within broader health systems [11, 35–37].

Of the CWW models examined in this review, most were implemented within or in collaboration with the health system. Noteworthy, no CWW models purported to be Indigenous-led, with most seeming to be operating from within Western, biomedical entities looking to enhance efficacy of existing mental health services in Indigenous contexts. A CWW model that integrates community engagement processes, may still be more susceptible to privileging Western, biomedical approaches over Indigenous approaches [55, 56]. Such dominance of Western, biomedical approaches can hamper a program’s ability to support Indigenous clients as they may need or wish to be supported. Examples might include program rigidity, where policies and processes are inflexible with strict power hierarchies and thus, programs cannot sufficiently bend to provide support as needed, where needed, and in a timely fashion [57]. Another might include the privileging of experts and methods deemed suitable by Western, biomedical understandings (such as counsellors, physicians, dieticians, security personnel, crisis intervention, etc.) over those valued and esteemed by Indigenous peoples (knowledge keepers, healers, Elders, ceremony, land-based activities, etc.). Other noted shortfalls of programs that are not Indigenous-led include mind-body dualism and forms of reductionism, individualism, or materialism [57]. These challenges related to power dynamics within CWW models were indeed discussed in the literature, primarily in reports of inequality between CWWs and clinicians or mainstream service providers.

Many articles underscored community engagement as important to the development and implementation of CWW models or programs; however, processes for community engagement were often not clearly stated. Many seemed to approach engagement by an initial consultation in co-development of the model/program, or as a single, post-implementation review. A significant number of articles (*n* = 11) did not report how communities were engaged at all. Instead, scholars urge for on-going Indigenous community participation and decision-making in across all stages (initiation, design, implementation, analysis, evaluation, and dissemination) of health and social service processes [58]. Community engagement is a crucial mechanism for upholding Indigenous self-determination and efficacy of mental wellness promotion programs [59]. Dominant Western approaches to mental wellness by themselves have largely failed at addressing the growing gap in mental health challenges between Indigenous and non-Indigenous populations [60]. Community-engaged models have a higher potential to be transformative, as they are underpinned by Indigenous perspectives, knowledge systems, and ways of understanding the world. These approaches also integrate Indigenous concepts of mental well-being and are firmly grounded in addressing community-identified and prioritized concerns [59].

We are aware of several additional examples of CWW models in Canada that were not captured in our search strategy. Local Indigenous workers with similar roles to CWWs are employed within the Brighter Futures and Building Healthy Communities Initiatives, National Native Alcohol and Drug Abuse Program (NNADAP), and National Aboriginal Youth Suicide Prevention Strategy (NAYSPS) [61]. These are long-standing initiatives employed in First Nations and Inuit communities in Canada to promote mental wellness. Local paraprofessional workers (referred to as community counselors, mental health workers or wellness workers, NNADAP or NAYSPS workers) within these programs deliver local workshops and related events intended to increase knowledge about mental health issues [62]. They also act as case managers, linking community members to resources and providing referrals to provincial, territorial, or external contracted services [62]. Nevertheless, these initiatives lack meaningful and effective integration or recognition within mainstream health and social services, and thus may not achieve the same successes observed within other CWW models examined in this review [63, 64]. In fact, in recognition of this lack of integration, the Government of Canada provides funding under the Health Services Integration Fund to projects to explicitly enhance the integration of these programs [65]. Additionally, while these above mentioned initiatives are deployed through local Indigenous governments under “self-government” agreements, Canada provides funding with strict stipulations and limitations [66]. Such arrangements grant little power and control to Indigenous communities, and has been criticized instead as merely “self-administration” [67]. More severe critiques highlight how these arrangements contribute to a continuance of neocolonial policies and practices, where Indigenous communities take over the performance of activities that realize the objectives of the colonialist state [67, 68]. Consequently, CWW models must consider who wields authority over program implementation and administration to fully leverage the advantages that come with truly community-engaged and Indigenous-led initiatives.

In this review, we identified several unique features of the roles and responsibilities as well as the training, education, and experience of CWWs that contributed to their success in promoting mental wellness. To supplement what we derived from the literature, the research team conducted a cursory search of CWW job postings and explored listed roles/duties and pre-service training, education, and experience requirements. Analysis of job postings revealed that roles and responsibilities largely aligned with the results of this review. Posted roles and responsibilities included case management; mental wellness intervention and counselling supports; supporting individuals, families, and the wider community; building relationships and collaborating with community, local Indigenous experts, and health and social service providers; providing cultural programming; and promoting cultural safety. Some noted that prospective candidates will be required to blend Indigenous and Western perspectives and work from a “two-eyed seeing” approach. “Two-eyed seeing,” as it is known in Mi’kmaq culture, is a framework in which practitioners are called upon “to see from one eye with the strengths of Indigenous ways of knowing, and to see from the other eye with the strengths of Western ways of knowing, and to use both of these eyes together, for the benefit of all” [69]. Contrary to findings in the literature, many job posting state a preference for candidates with formal diplomas or degrees in mental health, health related fields, or other relevant programs of study. However, they also emphasized experiential knowledge, knowledge and interest in traditional healing practices, and Indigenous cultures overall, fluency in or willingness to learn Indigenous languages, and knowledge of the impacts of colonization.

Findings from this rapid review revealed several distinct roles of CWWs. First, CWWs exhibit a strong emphasis on delivering strengths-based, family-centered mental wellness support, often facilitated through home visits. Family and household environments have been noted as an important avenue for mental wellness promotion [70–73]. This may be especially important in Indigenous contexts where colonization and on-going colonial policies have had considerable impacts on families [1, 74–80].

Furthermore, CWWs aim to promote holistic wellness of the individual while also considering relationships with the people and environments that surround them, especially relationships between family, households, the wider community, and the land [14]. The emphasis on interconnectedness and relationships by CWWs represents a vital element in promoting mental wellness within Indigenous contexts, which is often deficient in mainstream health and social services. Within an Indigenous worldview, “a person is viewed as an extension to, and is integrated with a family, community, tribe, and the creation/universe” [81]. This worldview emphasizes connectedness to a sense of self, to creation and the universe, and acknowledges the interdependence and interrelatedness of all things within the creation [81]. In Trauma and Recovery, Herman [82] outlines how connecting with others is vital to healing:

“The core experiences of psychological trauma are disempowerment and disconnection from others. Recovery, therefore, is based upon the empowerment of the survivor and the creation of new connections. Recovery can take place only within the context of relationships; it cannot occur in isolation” (p. 133).

Articles noted how CWW models place a high value on the lived-experience of workers, namely for their in-depth knowledge of community, cultural values, and their existing relationships within communities. However, articles noted how the strong bonds CWWs have with community can create challenges in carrying out their work. The suitability of individuals to provide mental wellness supports may vary depending on the dynamics within the community and longstanding relationship histories. Unfortunately, the presence of colonizing forces has led to a breakdown in connection and unity in many Indigenous communities. For instance, in Canada, the influence of Christian missionaries, the residential school system, and other on-going colonial processes have resulted in severed connections and unity at the community level [83, 84]. This is often manifested as jealousy, gossip, blame, shame, and community members undermining each other’s efforts to achieve wellness [83]. This phenomenon has been investigated thoroughly in recent years and has been coined as “lateral violence.” Lateral violence was defined by Middleton-Moz [85] as when members of an oppressed group feel powerless to fight back against a powerful oppressor and eventually turn their anger against each other in the form of “shaming, humiliating, damaging, belittling and sometimes violent behavior directed toward a member of a group by other members of the same group” (p. 116). Consequently, CWWs may encounter significant barriers in establishing the trust needed to effectively provide support to community members. In light of this, it is imperative for CWW models to take into account the unique local dynamics that can potentially create obstacles for CWWs and to work with communities to devise suitable strategies to effectively overcome them.

In addition, concerns of the maintenance of privacy and confidentiality may steer community members away from seeking support from CWWs. This has been a notable barrier reported in the literature around Community Health Worker models in other settings (for example, providing HIV-specific care in low- and middle-income countries) [4]. Perceived lack of confidentiality has been reported to have a negative impact on disclosure and trust, which is heightened for workers who are from and staying in the same communities that they serve [86–89]. Since CWWs work with people they share social spaces with and constantly interact with their families and neighbors, confidentiality is an ethical and practical imperative. Therefore, successful CWW models must ensure that workers are sufficiently supported in preserving the privacy and confidentiality of community members [4].

Integration of systems was both a characteristic of CWW models and a facilitator to their success. Mental health supports are usually found within parallel systems that focus on different areas of need and sometimes, these systems compete with one another for staff, resources, and funding [90]. This unfortunately leads to fragmented mental health service delivery. Integration of care and service delivery mitigates these challenges and ensures that the client’s needs are being addressed [91]. Integration is possible by having the CWW present in clinical settings and by having space for providers to share information about clients [91].

Many articles noted various systemic structural supports to promote supportive work environments for CWWs. None, however, referenced how different CWW models may be supporting CWW well-being and work/life balance via employee benefits. In Canada, for instance, many Indigenous workplaces provide benefits to employees beyond what is typically afforded in non-Indigenous workplaces. These benefits include paid leave to attend Treaty Days; to attend ceremony, cultural activities, or other spiritual events; for education pursuits; for community-wide bereavement; to vote in community elections; as well as recognition of Indigenous holidays [92, 93]. Some non-Indigenous institutions are starting to implement similar benefits for their Indigenous employees only [94, 95], whereas Indigenous organizations typically afford all employees the same benefits, regardless of Indigenous identity.

Lastly, many articles cited the challenges of recruiting and retaining Indigenous CWWs. A shift to recognition for the value of lived-experience of candidates paired with support for on-the-job training were reported as promising approaches to ameliorate this issue in the literature examined for this review. Other scholars have pointed to the absence of national Indigenous mental health strategies and subsequent inadequate funding as barriers to both recruiting Indigenous mental health professionals in Indigenous communities and to providing culturally competent mental health services [90].

This rapid review is limited in several ways. First, the rapid review process may not have captured all the relevant literature in this field. Rapid reviews aim to give an overview of a given field rather than systematically assessing each piece of evidence. They prioritize speed over thoroughness and often employ less exhaustive search strategies, which may lead to the omission of relevant literature [96]. Secondly, other than a cursory look at CWW-related job postings, this review is largely limited to public-facing, written reports of community wellness worker models. Further exploration into informal, oral, and/or non-publicly facing initiatives would be required to create a more comprehensive picture of existing CWW models. A key strength of this rapid review is the use of high-quality systematic review methodology, including the consideration of the quality of the included studies in formulating conclusions. Another important strength was the involvement of Indigenous community partners and that it was conducted with the intent of utilizing findings to inform a pilot CWW model in Regional Municipality of Wood Buffalo. This enhanced the focus of the review to gather relevant information and insights specifically tailored to the needs and goals of community and mental wellness service delivery.

## Conclusion

The pressing imperative to address the disproportionately elevated demand for mental wellness support in Indigenous communities worldwide has garnered significant recognition. This rapid review of the literature related to CWW models shows that these models present a promising means to begin to address this disproportionate burden. CWWs function as a critical link between Indigenous communities and mainstream health and social service providers. They fulfill distinctive roles in delivering heightened mental wellness support to community members, leveraging their strong ties to the community, close relationships with its members, deep knowledge of Indigenous culture, active advocacy for clients and the broader community, and adoption of holistic, family-centered, and strengths-based approaches. CWW models create space for the integrating of Indigenous and Western wellness perspectives. They incorporate “two-way” processes that emphasize the reciprocal, supportive, and mutually beneficial partnership between Indigenous agents and mainstream service providers. The positive outcomes of CWW models include, but are not limited to, early case detection, expansion of access, higher rates of engagement in services, improved patient adherence to treatment, enhanced efficacy of services, more thorough case documentation; reduced loss to follow-up, improved relationships and trust between clients and service providers, better management of crisis situations, greater cultural safety, and the bolstering of local capacity to promote mental wellness. CWW models employed several innovative structural solutions to bolster their efficacy, including via cross-functional and multidisciplinary teams, boosting supports and opportunities for workers, and efforts to improve system integration.

Nevertheless, substantial barriers to the success of CWW models endure. Such barriers include power imbalances and inequity between CWWs and mainstream service providers, a lack of role clarity, a lack of recognition from mainstream colleagues, mental wellness needs of workers, the high burden of mental health challenges within Indigenous communities, and more. Findings revealed that CWW models could benefit from improved efforts to ensure on-going Indigenous community participation and decision-making in across CWW model initiation, design, implementation, analysis, evaluation, and outcomes dissemination. Furthermore, it is imperative for governments and health authorities to embrace Indigenous-led CWW models as an integral component of comprehensive and culturally-sensitive mental health services for Indigenous communities. These commitments should be supported by mandatory or statutory funding mechanisms that guarantee Indigenous partners do not have to compete with mainstream service providers for resources.

While the purpose of this rapid review was to inform the development of an alternative service delivery model to support mental health and wellness among the Indigenous populations within the Regional Municipality of Wood Buffalo, we also hope the findings may be informative for other Indigenous communities and partners contemplating local mental wellness service delivery.

### Electronic supplementary material

Below is the link to the electronic supplementary material.

Supplementary Material 1

Supplementary Material 2

Supplementary Material 3

Supplementary Material 4

## Data Availability

Not applicable.

## Abbreviations

CWW: Community wellness worker
PICOD: Population, Intervention, Comparator, and Design
PRISMA: Preferred Reporting Items for Systematic Reviews and Meta-Analyses
