# Supplementary material for: Examining global Indigenous community wellness worker models: a rapid review
Source: Int J Equity Health. 2024 May 2;23:90. doi: 10.1186/s12939-024-02185-5 (PMC11065687; doi:10.1186/s12939-024-02185-5)
Supplement: Supplementary file 2 — Supplementary Material 2 [file 12939_2024_2185_MOESM2_ESM.docx]

**Additional File 2: Academic Literature Search Strategy and Terms by Database**

**Methods: Search**

A search was executed by an expert searcher/health librarian (SC) on the following databases: OVID Medline and Global Index Medicus using controlled vocabulary (eg: MeSH, etc) and key words representing the concepts “Indigenous" and "community health workers". Search filters for applied for Indigenous groups^1,2,3,4,5,6,7,8,9,10,11^. No limits were applied. Databases were searched from inception to January 20, 2022. Results (590) were exported to COVIDENCE review management software, where duplicates (61) were removed. Detailed search strategies and terms are available in Appendix 1 below.

**References:**

1. Campbell, Sandy, Marlene Dorgan and Lisa Tjosvold. Filter to Retrieve Studies Related to Indigenous People of Canada the OVID Medline Database. John W. Scott Health Sciences Library, University of Alberta. Rev. October 4, 2021. <https://docs.google.com/document/d/1XqpWHN7hrFIyNwaqucRFRXaCnBOaeshFw4SR31Uxyek/edit>
2. Campbell, Sandy, Marlene Dorgan and Lisa Tjosvold. Filter to Retrieve Studies Related to Indigenous People of Canada the EBSCO CINAHL Database. John W. Scott Health Sciences Library, University of Alberta. Rev. January 20, 2022. [https://docs.google.com/document/d/17EZUiMOFKrhjn9XnOBZFjazCf8m7PCnKT0IeAp3Bkmg/edit#](https://docs.google.com/document/d/17EZUiMOFKrhjn9XnOBZFjazCf8m7PCnKT0IeAp3Bkmg/edit)
3. Campbell, SM. Filter to Retrieve Studies Related to Indigenous People of the United States from the OVID Medline Database. John W. Scott Health Sciences Library, University of Alberta. Rev. Sept 19, 2021. <https://docs.google.com/document/d/118tP1FvgQ1hRROjI1QroLjM-u8WN_uS6Nafis6s37jk/edit>
4. Campbell, SM.. Filter to Retrieve Studies Related to Indigenous People of the United States from the EBSCO CINAHL Database. John W. Scott Health Sciences Library, University of Alberta. Rev. March 21, 2021. <https://docs.google.com/document/d/118tP1FvgQ1hRROjI1QroLjM-u8WN_uS6Nafis6s37jk/edit>
5. Campbel SM. A Filter to Retrieve Studies Related to Indigenous People of Australia and the Torres Strait Islands from the Ovid MEDLINE Database. John W. Scott Health Sciences Library, University of Alberta. Rev. 01_20_2022. [https://docs.google.com/document/d/15g260L_hRKgYCh-iygS_QHI1cvGlSsphd5XwlrSk2Tg/edit#](https://docs.google.com/document/d/15g260L_hRKgYCh-iygS_QHI1cvGlSsphd5XwlrSk2Tg/edit)
6. Campbell, SM. A Filter to Retrieve Studies Related to Indigenous People of Australia and the Torres Strait from the EBSCO CINAHL Database. John W. Scott Health Sciences Library, University of Alberta. Rev. 01_20_2022. [https://docs.google.com/document/d/1pMMnvjKyJwy73FKie34BZLryKT5t-4izumfQosuJn8Y/edit#](https://docs.google.com/document/d/1pMMnvjKyJwy73FKie34BZLryKT5t-4izumfQosuJn8Y/edit)
7. Campbell SM. A Filter to Retrieve Studies Related to Maori People from the Ovid MEDLINE Database. John W. Scott Health Sciences Library, University of Alberta. Rev. 01_20_2022. [https://docs.google.com/document/d/1AyTKFnQtJ3THHxporefpSFovNox_G4HeqvIqVdgBvaI/edit#](https://docs.google.com/document/d/1AyTKFnQtJ3THHxporefpSFovNox_G4HeqvIqVdgBvaI/edit)
8. Campbell SM. A Filter to Retrieve Studies Related to Maori People from the Ovid MEDLINE Database. John W. Scott Health Sciences Library, University of Alberta. Rev. 01_20_2022. [https://docs.google.com/document/d/1AyTKFnQtJ3THHxporefpSFovNox_G4HeqvIqVdgBvaI/edit#](https://docs.google.com/document/d/1AyTKFnQtJ3THHxporefpSFovNox_G4HeqvIqVdgBvaI/edit)
9. Campbell, SM. A Filter to Retrieve Studies Related to Sami People from the Ovid MEDLINE Database. John W. Scott Health Sciences Library, University of Alberta. Rev. 05_01_2020. [https://docs.google.com/document/d/1w_lnUXzOZF3cYdwyklRs_ADg0aDQeUY-EfcZvy9Gayw/edit#](https://docs.google.com/document/d/1w_lnUXzOZF3cYdwyklRs_ADg0aDQeUY-EfcZvy9Gayw/edit)
10. Campbell SM. A Filter to Retrieve Studies Related to Sami People from the EBSCO CINAHL Database. John W. Scott Health Sciences Library, University of Alberta. Rev. 01_20_2022. <https://docs.google.com/document/d/1zWCI5kcvGLFtoFI8eP-0Zoyuz73xpMTi5ryQrG1HiW8/edit>
11. Campbell, SM. Filter to Retrieve Studies Related to Indigenous People of Mexico in the EBSCO CINAHL Database. John W. Scott Health Sciences Library, University of Alberta. Rev. January 20, 2022 <https://docs.google.com/document/d/1QwvbY6HSvL9XhixdeQBbjFrXFNjJBG3A6zmUDIM7IGY/edit>

**Appendix 1: Detailed Search Strategy & Terms**

**Medline**

| # | Search Statement | Results |
| --- | --- | --- |
| 1 | (Indigenous mental health adj6 (worker* or peer* or liaison* or helper* or agent* or advocat*)).mp. | 7 |
| 2 | "Community Health Workers"/ or ("community health worker*" or "local health worker*" or "community health agent*" or "community health aide*" or "community health liaison*" or "community health guide*" or "community health navigator*" or "lay health worker*" or "lay health advocat*" or "community led").mp. or CHW.ti,ab. | 10359 |
| 3 | (exp Indians, North American/ or exp Inuits/ or exp Health Services, Indigenous/ or exp Ethnopharmacology/ or Athapaskan.mp. or Saulteaux.mp. or Wakashan.mp. or Cree.mp. or Dene.mp. or Inuit.mp. or Inuk.mp. or Inuvialuit*.mp. or Haida.mp. or Ktunaxa.mp. or Tsimshian.mp. or Gitsxan.mp. or Nisga'[a.mp](http://a.mp/). or Haisla.mp. or Heiltsuk.mp. or Oweenkeno.mp. or Kwakwaka'[wakw.mp](http://wakw.mp/). or Nuu chah [nulth.mp](http://nulth.mp/). or Tsilhqot'[in.mp](http://in.mp/). or Dakelh.mp. or Wet'suwet'[en.mp](http://en.mp/). or Sekani.mp. or Dunne-za.mp. or Dene.mp. or Tahltan.mp. or Kaska.mp. or Tagish.mp. or Tutchone.mp. or Nuxalk.mp. or Salish.mp. or Stl'[atlimc.mp](http://atlimc.mp/). or Nlaka'[pamux.mp](http://pamux.mp/). or Okanagan.mp. or Sec [wepmc.mp](http://wepmc.mp/). or Tlingit.mp. or Anishinaabe.mp. or Blackfoot.mp. or Nakoda.mp. or Tasttine.mp. or Tsuu T'[inia.mp](http://inia.mp/). or Gwich'[in.mp](http://in.mp/). or Han.mp. or Tagish.mp. or Tutchone.mp. or Algonquin.mp. or Nipissing.mp. or Ojibwa.mp. or Potawatomi.mp. or Innu.mp. or Maliseet.mp. or Mi'[kmaq.mp](http://kmaq.mp/). or Micmac.mp. or Passamaquoddy.mp. or Haudenosaunee.mp. or Cayuga.mp. or Mohawk.mp. or Oneida.mp. or Onodaga.mp. or Seneca.mp. or Tuscarora.mp. or Wyandot.mp. or Aboriginal*.mp. or Indigenous*.mp. or Metis.mp. or red [road.mp](http://road.mp/). or "on reserve".mp. or [off-reserve.mp](http://off-reserve.mp/). or First Nation.mp. or First Nations.mp. or Amerindian.mp. or (urban adj3 (Indian* or Native* or Aboriginal*)).mp. or [ethnomedicine.mp](http://ethnomedicine.mp/). or country food*.mp. or residential school*.mp. or ((exp Medicine, Traditional/ or traditional medicine*.mp.) not Chinese.mp.) or exp Shamanism/ or shaman*.mp. or traditional heal*.mp. or traditional food*.mp. or medicine [man.mp](http://man.mp/). or medicine [woman.mp](http://woman.mp/). or autochtone*.mp. or (Native* adj1 (man or men or women or woman or boy* or girl* or adolescent* or youth or youths or person* or adult or people* or Indian* or Nation or tribe* or tribal or band or bands)).mp.) and (exp Canada/ or (Canad* or British Columbia or Colombie Britannique or Alberta or Saskatchewan or Manitoba or Ontario or Quebec or Nova Scotia or New Brunswick or Newfoundland or Labrador or Prince Edward Island or Yukon Territory or NWT or Northwest Territories or Nunavut or Nunavik or Nunatsiavut or NunatuKavut).mp.) | 8259 |
| 4 | Indigenous People/ or American Indian/ or Canadian Aboriginal/ or Eskimo/ or Inuit/ or Indigenous Health Services/ or ("A' ani" or Absaroka or Haaninin or Atsina or "Gros Ventre" or Acopsel or Tlacopsel or Lacopsel or Ahtna or Ahtena or Akenitsi or Occaneechi or Akokisa or Horcoquisa or Orcoquizas or Aleut or Unangax or Unangan or Alibamu or "Alabama Alsea" or Alutiiq or Sugpiag or Amahami or Awaxawi or Androscoggin or Arosaguntacook or Ameriscoggin or Anishinaabeg or Chippewa or Anihsinape or Saulteaux or Apalachee or Aranama or "Texan Coahuilteca" or Tamique or Arikara or Sahnish or Arickaree or Adakadaho or Assiniboine or Hohe or Nakota or Nakoda or Nakona or "Atsa' Kudok-wa" or Awatixa or Bannock or "Snake Indian*" or Bidai or Quasmigdo or Biloxi or Blackfoot or Niitsitapi or Sikasikaitsitapi or Cahto or Kaipomo or Cahuilla or Ivilyuqaletem or Ivilyuat or Catawba or Inna or Iswa or Chemehuevi or Chickasaw or "Chilula Chimakum" or Aqokulo or Chimariko or Chiricahua or Tsokanende or Chitimacha or Chetimachan or Sitimacha or Chowanoke or Roanoke or Chumash or Ciboney or "Taino Ciwat" or Clatsop or Coos or Coosa or Uchis or Chiaha or Coste or Talisi or Coquille or Kokwell or Coso or Cowlitz or Taitnapam or "Crow Nation" or "Cui Ui Ticutta" or Cupeno or Kuupangaxwichem or Cupa or "Cup' ig" or Nunivak or "Dakota Oyate" or Lakota or Nakota or Santee or Teton or Sioux or Deadose or "Deg Xina" or "Deg Xit' an" or Kaiyuhkhotana or "Deg Hit' an" or "Dena' ina" or Tanaina or "Dichinanek' Hwt' ana" or "Upper Kuskokwim Athabascan*" or Kolchan or Goltsan or "Tundra Kolosh" or "Do lkabya" or Duwamish or Esselen or Eyak or "Gidi' tikadi" or Guwevkabaya or "Gwich' in" or Kutchin or Haida or Xaadas or Xaat or Halchidhoma or Havasupai or "Green Water People" or Hiratsa or Hiraaca or "Ho-chaaqa" or Winnebago or Holikachuk or Innoko or "Tlegon-khotana" or Hopi or "Houma-Louisiana" or Huaco or Waco or Hualapai or Hupa or Natinixwe or "Natinook-wa" or "Hwech' in" or Hankutchin or "Iroquois Confederacy" or "Hodinoso ni" or "Illinois Confedera*" or Ilinoweg or Illini or Inupiat or Inuit or Ioway or Baxoje or Jicarilla or Juaneno or Acjachemen or Jumano or Kalapuya or Clackama or Kalispel or "Pend d' Oreilles" or Qlispe or Karuk or Karok or "Chum-ne" or Katkoc or Kansa or Kanza or Kawaiisu or Nuwa or Kennebec or "Kinipekw Kittitas" or Klickitat or "Qwu' lh-hwai-pum" or "Awi-adshi" or Mahane or Wahnookt or "Koa' aga' itoka" or Keresan or Kichai or Kitsai or Keechi or "K' itaish" or Kiowa or Gaigwu or Cauigu or Kutjau or "Kwu-da" or "Tep-da" or Kitanemuk or Kittitas or Klickitat or "Qwu' lh-hwai-pum" or "Awi-adshi" or Mahane or Wahnookt or "Koa' aga' itoka" or Konkow or "Koop Ticutta" or Koyukon or Ktunaxa or Kootenai or Flathead or Kucadikadi or "Kotsa' va" or Kumeyaay or "Tipai-Ipai" or Kamia or Diegueno or Kwapa or Cocopah or Cucapa or "Xawitt kwnchawaay" or Lassik or Lenape or "Leni-Lenape" or Lipan or Luiseno or Payomkawichum or Madqwadabaya or "Desert Yavapai" or Mahican or Mohicans or Makah or Makuhadokado or Maliseet or Wolistoqiag or Manahoac or Mahock or Meipontsky or Mandan or Mattole or "Bear River" or "Tul' bush" or "Ni' ekeni" or Meherrin or Menominee or Mackinac or Mescalero or Myaamiaki or Kickapoo or Twigtwee or Missouria or Miwok or Miwuk or Moadokado or Modoc or Mohave or "Aha Makhav" or Mohawk or "Kaneng' hega" or Molala or Molale or Molele or Nyyhmy or Moosonee or "Moose Cree" or Monsonis or Multnomah or Chinook or Nabedache or Nabaydacu or Wawadishe or Nabiltse or Dakubetede or "Nacho Nyak Dun" or Tutchone or Nacono or "Na' isha" or Nanticoke or Navajo or Ndee or Nial or Niimiipu or "Nez Perce" or Watapala or Watapahlute or Nisenan or Nisqually or Nomlaki or Noamlakee or "Central Wintun" or Nongatl or Nottoway or Cheroenhaka or "Northern Cheyenne" or Ohlone or Costanoan or Omaha or "O' odham" or Pima or Papago or Osage or Otoe or Otse or "Ozav Dika" or Palus or Passamaquoddy or Pestomuhkati or Patiri or Petaros or Pastia or Patwin or "Southern Wintun" or Panis or Skidi or Pedee or Penobscot or "Petun Piipaash" or "Kokmalik' op" or Piscatawa or Doeg or Conoy or "Pit River" or Pomo or Kashaya or Ponca or Ponka or Pottawatomi or Bodewadmik or Powhatan or Puyallup or Spuyalepabs or Quapaw or Ugahxpa or Quechan or Yuma or Kwtsaan or Quileute or Salinan or Saponi or Monacan or Sapon or "Eastern Blackfoot" or Christanna or Sawawatodo or Serrano or Taaqtam or "Maarenga' yam" or Yuhaviatam or Shasta or Chasta or Sasti or Shoshone or Siletz or Sinkine or Sinkyone or "Siuslaw Umpqua" or Skitswish or "Schitsu' umash" or Snohomish or Snuqualmi or Sokoki or Missiquoi or Stillaguamish or Stoluckwamish or Suquamish or Sutaio or Swinomish or Skagit or Syilx or Okanagan or Sotaae or "Taga Ticutta" or Takelma or Dagelma or Taltushtuntede or Galice or "Tanan Gwich' in" or Taos or Taovaya or Tataviam or Alliklik or Tawakoni or Tahuacano or Tenino or Thawikila or Hathawekela or "Fort Ancient" or Tigua or Tillamook or Nehalem or Timbisha or Panamint or Timpanogos or Tlingit or "Toi Ticutta" or Tolowa or "Talawa Dini' " or Tongva or Gabrieleno or Fernandeno or Tobikhar or Tonkawa or Ticanwatic or Tsikip or Appalousa or Opelousa or Tsitsistas or Tubatulabal or Tukabatchee or Tuscarora or Tomahittan or Kuskarawock or Tutelo or Tutero or Totteroy or Tutera or Yusan or Tututni or Umatilla or Umpqua or Waccamaw or Waxmaw or Wadatika or "Harney Valley Paiute" or Wailiki or Waluulapam or "Walla Walla" or Walpapi or Huipui or Wampanoag or Massasoit or Wanapum or Wappo or Washoe or Wichita or Willapa or Kwalhioqua or "Wi pukba" or "Verde Valley Yavapai" or Wintu or "Northern Wintun" or Wiyot or "Wee' at" or Weyet or Yakama or "Yamosopo Tuviwarai" or Yaqui or Yoeme or Yatasi or Yattasih or "Yavbe' " or "Yavapai" or "Ysleta del Sur" or Yojuane or Yokuts or Mariposa or Yuki or Yupighyt or "Yup'ik" or Yupik or Yurok or "Olekwo'l" or Zuni).mp. or ((Applegate or Delaware or Iowa or Ishak or Kaw or Kato or Spokane or Miami or Arkansas or Tali or Tunica or Han or Pawnee or "Coeur D' Alene" or Piscataway or Ree or Tula) adj3 (reservation* or nation or people or peoples or population or man or men or woman or women or child* or youth* or elder or elders or communit* or tribe or tribes or tribal or Indian*)).mp. | 32357 |
| 5 | (Saami or Sampi or (Sami not Ulus) or Samis or Southernsami* or Umesami* or Pitesami* or Lulesami* or Northernsami* or Enaresami* or Kolasami* or Lapp or Lapps or Lappish or Lappland or (Lapland* not longspur) or Lappalainen* or Saamelainen* or reindeer herd* or reindeer culture* or reindeer pastoral* or Lappbys or Samebys or reinbeitesdistrikt or paliskunta or siida).mp. or (((Fennoscandia or Finnmark or Scandinavia or Nordic or Sweden or Norway or Finland or Swedish or Finnish or Norwegian or Norge or Svensk* or Suomi or Barents Region or (Kola not (garcinia or gotu)) or Arctic Europe* or Polar Europe* or North* Europ*).mp. or Finland/ or Norway/ or Sweden/) and ((traditional adj3 (food* or heal* or medicine* or shaman*)) or (Indigenous* adj3 (people* or person* or mother* or father* or parent* or child* or boy or boys or girl* or youth* or healer* or patient* or famil* or herder*))).mp.) | 1506 |
| 6 | ((exp australia/ or (australia* or northern territory or tasmania or new south wales or Victoria or queensland).ti,ab.) and (exp oceanic ancestry group/ or aborigin*.ti,ab. or indigenous.ti,ab.)) or torres strait islander*.ti,ab. | 11453 |
| 7 | (maori or tangata whenua or mauori or moriori or mauri or ((New Zealand or Christchurch or Aukland) and (indigenous or aboriginal or "first people*" or shaman* or tribe or tribes or tribal or clan or clans))).mp. or (exp New Zealand/ and (indigenous or aboriginal or "first people*" or shaman* or tribe or tribes or tribal or clan or clans).mp.) or ((exp New Zealand/ or (New Zealand or Aukland).mp.) and (exp Oceanic Ancestry Group/ or (aborig* or Indig*).ti,ab.)) | 5238 |
| 8 | (Abipon or Achuar or Achuagua or Akawaio or Amarizana or Andoque or Akawaio or Akuriyo or Anauya or Araona or Arawak or Ayamn or Aguaruna or Amahuaca or Amarakaeri or Andoa or Arabela or Arawak or Arhuaco or Ashaninca or Asheninca or Atsahuaca or Aymara or Ayoreo or Bakairi or Baniva or Barasana or Baniwa or Baure or Bororo or Cabiyari or Cacataibo or Caquinte or Cacua or Cahuarano or Caiua or Camara Indians or Camaracoto or Camsa or Canamari or Candoshi or Canela or Canichana or Capanahua or Carapana or Cariay or Carib or Carijona or Carutana or Cashibo or Cashinahua or Cawishana or Cavinena or Caxuiana or Cayuvava or Chontaquiro or Cocama or Cubeo or Curipaco or Chacobo or Chaima or (Chana not striatus) or Chapacura or Charrua or Chimila or Chitonahua or Chorote or Chipaya or Chiquitano or Chulupi or Carare or Coconuco or Cofan or Coreguaje or Coyaima or Chamacoco or Chamicuro or Chayahuita or Cocama or Culina or Culino or Cubeo or Cuiba or Cuiva or Cumanagoto or Curripaco or Deni or Desano or Embera or Guarani or Guajajara or Guana or Guanano or Guarayo or Guarayu or Guahibo or Guajiro or Guambiano or Guanano or Guayabero or Guarequena or Guinao or Guana or Gayon or Guahibo or Hixkaryana or Huachipairi or Huambisa or Huarayo or Iauanaua or Ikpeng or Ingariko or Irantxe or Itonama or Inapari or Iquito or Isconahua or Jumana or Japreria or Jirajara or Juruti or Jaqaru or Jebero or Kadiweu or Kaingang or Kamayura or Karaja or Karipuna or Kariri or Katukina or Kaxarari or Kayabi or Kayapo or Kuikuro alapalo or Kulina or Kaiwa or Kallawaya or Kogui or Kuna or Kaweskar or Lule or Macuna or Maipure or Mapuche or Mataco or Mocovi or Machinere or Machinerev or Machiguenga or Macushi or Macuna or Madi or Malayo or Mamainde or Manao or Mandauaca or Mandawaka or Mapidian or Mapuche or Mapidian or Maquiritare or Maquiritari or Maragua or Marawan or Mariate or Marubo or Mastanahua or Matipuhy or Matis or Matses or Mawakua or Mawakwa or Maxakali or Mehinaku or Miranha or Moronawa or Munduruku or Mataco or Movima or Muellama or Muinane or Mapoyo or Mashco Piro or Matses or Muniche or Nambikwara or Nocaman or Nuquini or Nomatsiguenga or Nanti or Ocaina or Omagua or Orejon or Opon or Pacahuara or Paez or Paicone or Palicur or Panare or Pano or Paresi or Paumari or Pemon or Pilaga or Puelche or Pauna or Pauserna or Piapoco or Piraha or Piratapuyo or Pisabo or Piaroa or Pijao or Piratapuyo or Paraujano or Pemon or Pemono or Piapoco or Puinave or Patamona or Poyanawa or Puinave or Puquina or Quechua or Quichua or Retuara or Resigaro or Reyesano or Sabanes or Saliba or Saluma or Sarave or Secoya or Selknam or Sensi or Shaninawa or Shapra or Sharanahua or Shebayo or Shiwiar or Shikiana or Sikiana or Siriono or Sinsiga or Siona or Suruwaha or Tacano or Tamanaco or Tiahuanaco or Tariano or Tehuelche or Tariano or Tatuyo or Tembe or Terena or Telembi or Ticuna or Ticuna or Tiriyo or Tiwanaku or Tiwanaku or Torom or Totoro or Tsimane or Tuberao or Tucano or Tunebo or Tuxinawa or Tuyuca or Uainuma or Urarina or Vilela or Waimaha or Waiampi or Waiwai or Wapishana or Waraiku or Warekena or Waura or Wayampi or Wayana or Wirina or Waimaha or Waunana or Wiwa or Warao or Wayuu or Witoto or Xavante or Xipaya or Xiriana or Xokleng or Yabaana or Yaminawa or Yaminahua or Yaruma or Yawalapiti or Yuracare or Yabarana or Yavitero or Yine or Yamana or Yaghan or Yucuna or Yurumangui or Yukpa or Yanesha or Yoranahua or Yagua or Yaminahua or Zaparo or Zamuco or ((Inga or Maca or Leco or Mojo or Uro or Maco or Lengua or Toba or Zoe or Ona or Catio or Passe or Bari or Awa or Bora or Bara or Remo or Pano or Sape) adj3 (Indians or Indian or Indigenous or Amerindian* or Aborigin* or people or peoples or women or men or woman or man or child* or youth or youths or baby or babies or tribe or tribes or tribal or shaman* or native or traditional)) or Trio Indians or More Indians or Bare Indians).mp. or ((Indian* or Amerindian, or Aboriginal* or indigenas or Indigenous) and (Argentin* or Bolivia* or Brazil* or Chile* or Colombia* or French Guiana* or Guyana* or Peru or Paraguay or Uruguay or Venezuela or Amazon or Amazons or Amazonia or Andes or Andean)).tw. or exp Indians, South America/ [South American Indigenous] | 9135 |
| 9 | (Acatec or Aguacateco or Amuzgo or Bokota or Boruca or Bribri or Bri Bri or Buglere or Cabecar or Cakchiquel or Changuena or Chatino or Chiapanec or Chicomuceltec or Chinantee or Chocho or Cholti or Ch'olti' or Ch'olti'an or Chontal or Chorotega or Chorti or Chuj or Chumbia or Corobici or (Cueva not Spain) or Cuicatec or Cuitlatee or Cuytec or Dorasque or Embera or Garifuna or Guatuso or Guaymi or Guaymis or Guetar or Huastec or Huave or Huetar or Itzaj or Ixil or Jacalteco or Jonaz or Kanjobal or Kekchi or Kuna or Maleku or Mangue or Matambu or Matlatzinca or Mazahua or Motozintlec or Mayan or Mayangna or Miskito or Mixtec or Mopan or Nahua or Nahuatl or Ngabe or Otomi or Pantec or Paya or Popoloca or Popoloc or Poqomam or Poqomchi or Q'eqchi' or Quiche or Quitirrisi or Sacapulteco or Sipacapense or Subtiaba or Tacaneco or Tarasco or Tamaulipec or Tamazultec or Tecoxquin or Tectiteco or Tecual or Tecuexe or Tepehura or Tepuztecor or Teribe or Terraba or Totonac or Trique or Tzeltal or Tzotzil or Tzutujil or Ulwa or Uspantec* or Uspanteko or Voto or Xinca or Waunana or Wounaan or Yucatec or Zapotec or Zoque or ((Costa Rica* or Hondura* or Nicaragua* or Panama* or Guatemala* or Achi or Belize or Belizean* or Maya* or Mixe or Pame or Pipil or Pech or Chol or Ch'olan or Cora or Cuna or Mam or Rama) adj5 (Indian or Indians or Amerindian*OR Amerindio* or Aboriginal* or Indigenous or Indigena* or Aborigen* or Mestizo or tribe or tribes or tribal or traditional medicine* or shaman*))).tw. not (Mexico.mp. or exp Mexico/) [Central America - Indigenous] | 1076 |
| 10 | ((((Acatec or Aguactec or Amuzgo or Chatino or Chiapaneca or Chichimeca or Chicomuceltec or Chinantec* or Chocho or Chontal or Chuj or Cochimi or Cocopa or Cuicatec* or Guarijio or Haurijio or Huastec* or Huave or Huichol or Ixcatec* or Jacaltec* or Kanjobal or Kaqchikel or Kekchi or K'iche' or Kickapoo or Kikapu or Kiliwa or Kumiai or Lacandon or Matlatzinca or Mazahua or Mazatec* or Mixe or Mixtec* or Mocho or Motocintleco or Nahua* or Oaxaca or Ocuiltec or Opata or Otomi or Paipai or Pame or Papago or Pima Bajo or Popoloca or Popoluca or (Purepecha not Echeveria) or Raramuri or Tabasco Chontal or Tacaneco or Tacuate or Tarahumara or Tectiteco or Teenek or Tenek or Tepehua or Tepehuan or Tlahuica or Tlapanec or Tohono O'odham or Tojolabal or Toltec or Totonac or Trique or Triqui or Tsotsil or (Tubar not ("tubar pregnancy" or "tubar sterility")) or Tzeltal or Tzotzil or Wixarika or Yaqui or Yucatec Maya or Zapotec or Zoque).tw. or ((Cora or Mame or Maya or Mayos or Mayo or Mam or Chol or Ch'ol or Ixil or Seri) adj3 (Indian* or tribe* or tribal or people* or person or elder or youth or child or children or men or men or man or woman or women or adolescent)).mp. or (((Mexico not New Mexico) or Aguascalientes or Baja California or "Los Cabos" or "La Paz" or Loreto or Campeche or Chiapas or Chihuahua or Coahuila or Colima or Manzanillo or Distrito Federal or Durango or "Estado de Mexico" or Guanajuato or "Leon San Miguel de Allende" or Guerrero or Acapulco or Hidalgo or Jalisco or Puerto Vallarta or Guadalajara or Michoacan or Morelia or Morelos or Nayarit or Nuevo Leon or Oaxaca or Huatulco or Puerto Escondido or Puebla or Queretaro or Quintana Roo or Cancun or Cozumel or San Luis Potosi or Sinaloa or Sonora or Tabasco or Tamaulipas or Tlaxcala or Veracruz or Yucatan or Merida or Zacatecas or Zenacantan* or Zenacanteco*) adj7 ((pre Columbian adj3 culture*) or (preColumbian adj3 culture*) or Indian or Indians or Aboriginal* or Indigen* or First Peoples or Native Mexican* or indos mexicano or pueblos indigenas or shaman* or tribe or tribal or tribes or Amerindian* or traditional or Mesoamerindian*)).tw.) not ((Blinder adj2 Oaxaca) or Guatemala or Pepper Huasteco Virus or opata m85 or polar or cladoceran*).mp.) or ((Indians, North American/ or ((pre Columbian adj3 culture*) or (preColumbian adj3 culture*) or Indian or Indians or Aboriginal* or Indigen* or First Peoples or Native Mexican* or indos mexicano or pueblos indigenas or shaman* or h'iloletik or curander* or curandeiro or yerberos or herbalist* or hueseros or parteras or oracionistas or tabaqueros or ayahausqueros or peyoteros or sobadores or espiritualistas or tribe or tribal or tribes or Amerindian* or traditional or Mesoamerindian*).mp.) and (Mexico/ or (Mexican or mexico or mexico's or mexicano or mexicali).mp.))) not (exp animals/ not (exp humans/ and exp animals/)) [Mexico-Indigenous] | 6208 |
| 11 | 3 or 4 or 5 or 6 or 7 or 8 or 9 or 10 | 67538 |
| 12 | 1 or (2 and 11) [CHW + Indigenous] | 384 |

| EBSCO CINAHL Searched January 20, 2022  Limiters/Expanders Find all my search terms   \| **#** \| **Query** \| **Results** \| \| --- \| --- \| --- \| \| S1 \| ( ( Athapaskan or Saulteaux or Wakashan or Cree or Dene or Inuit or Innu or Inuk or Inuvialuit* or Haida or Ktunaxa or Tsimshian or Gitsxan or “Nisga'a” or Haisla or Heiltsuk or Oweenkeno or “Kwakwaka'wakw” or “Nuu chah nulth” or “Tsilhqot'in” or Dakelh or “Wet'suwet'en” or Sekani or “Dunne-za” or Dene or Tahltan or Kaska or Tagish or Tutchone or Nuxalk or Salish or “Stl'atlimc” or “Nlaka'pamux” or Okanagan or “Sec wepmc” or Tlingit or Anishinaabe or Blackfoot or Nakoda or Tasttine or “Tsuu T'inia” or “Gwich'in” or Han or Tagish or Tutchone or Algonquin or Nipissing or Ojibwa or Potawatomi or Innu or Maliseet or “Mi'kmaq” or Micmac or “Mic mac” or Passamaquoddy or Haudenosaunee or Cayuga or Mohawk or Oneida or Onodaga or Seneca or Tuscarora or Wyandot or Aboriginal* or Indigenous* or Metis or “red road” or "on reserve" or “off reserve” or “First Nation” or “First Nations” or Amerindian or (urban N3 (Indian* or Native* or Aboriginal*)) or ethnomedicine or “country food*” or “residential school*” or ("traditional medicine*" ) not Chinese ) or or shaman* or “traditional heal*” or “traditional food*” or “medicine man” or “medicine woman” or autochtone* or (Native* N1 (American* or man or men or women or woman or boy* or girl* or adolescent* or youth or youths or person* or adult or people* or Indian* or Nation or Nations or tribe* or tribal or band or bands or elder or elders or patient*)) ) ) AND ( ( (MH “Canada+”) or (Canad* or “British Columbia” or “Colombie Britannique” or Alberta or Saskatchewan or Manitoba or Ontario or Quebec or “Nova Scotia” or “New Brunswick” or Newfoundland or Labrador or “Prince Edward Island” or “Yukon Territory” or NWT or “Northwest Territories” or Nunavut or Nunavik or Nunatsiavut or NunatuKavut)) ) \| 1,182 \| \| S2 \| (MH "Aboriginal Canadians") OR (MH "Indigenous Peoples") OR (MH "First Nations of Canada") OR (MH "Arctic Peoples") OR (MH "Inuit") OR (MH "First Nations of Australia") OR (MH "Aboriginal Australians") OR (MH "Torres Strait Islanders") OR (MH "Maori") OR (MH "Native Americans") OR (MH "Alaska Natives") \| 21,924 \| \| S3 \| (MH "Indigenous Health") \| 1,442 \| \| S4 \| (MH "Health Services, Indigenous") \| 2,657 \| \| S5 \| (MH "Medicine, Native American Traditional") OR (MH "Shamanism") \| 291 \| \| S6 \| ("A' ani" or Absaroka or Haaninin or Atsina or "Gros Ventre" or Acopsel or Tlacopsel or Lacopsel or Ahtna or Ahtena or Akenitsi or Occaneechi or Akokisa or Horcoquisa or Orcoquizas or Aleut or Unangax or Unangan or Alibamu or "Alabama Alsea" or Alutiiq or Sugpiag or "Pacific Yupik" or Amahami or Awaxawi or Androscoggin or Arosaguntacook or Ameriscoggin or Anishinaabeg or Chippewa or Anihsinape or Saulteaux or Apalachee or Aranama or "Texan Coahuilteca" or Tamique or Arikara or Sahnish or Arickaree or Adakadaho or Assiniboine or Hohe or Nakota or Nakoda or Nakona or "Atsa' Kudok-wa" or Awatixa or Bannock or "Snake Indian*" or Bidai or Quasmigdo or Biloxi or Blackfoot or Niitsitapi or Sikasikaitsitapi or Cahto or Kaipomo or Cahuilla or Ivilyuqaletem or Ivilyuat or Catawba or Inna or Iswa or Chemehuevi or Chickasaw or "Chilula Chimakum" or Aqokulo or Chimariko or Chiricahua or Tsokanende or Chitimacha or Chetimachan or Sitimacha or Chowanoke or Roanoke or Chumash or Ciboney or "Taino Ciwat" or Clatsop or Coos or Coosa or Uchis or Chiaha or Coste or Talisi or Coquille or Kokwell or Coso or Cowlitz or Taitnapam or "Crow Nation" or "Cui Ui Ticutta" or Cupeno or Kuupangaxwichem or Cupa or "Cup' ig" or Nunivak or "Dakota Oyate" or Lakota or Nakota or Santee or Teton or Sioux or Deadose or "Deg Xina" or "Deg Xit' an" or Kaiyuhkhotana or "Deg Hit' an" or "Dena' ina" or Tanaina or "Dichinanek' Hwt' ana" or "Upper Kuskokwim Athabascan*" or Kolchan or Goltsan or "Tundra Kolosh" or "Do lkabya" or Duwamish or Esselen or Eyak or "Gidi' tikadi" or Guwevkabaya or "Gwich' in" or Kutchin or Haida or Xaadas or Xaat or Halchidhoma or Havasupai or "Green Water People" or Hiratsa or Hiraaca or "Ho-chaaqa" or Winnebago or Holikachuk or Innoko or "Tlegon-khotana" or Hopi or "Houma-Louisiana" or Huaco or Waco or Hualapai or Hupa or Natinixwe or "Natinook-wa" or "Hwech' in" or Hankutchin or "Iroquois Confederacy" or "Hodinoso ni" or "Illinois Confedera*" or Ilinoweg or Illini or Inupiat or Inuit or Ioway or Baxoje or Jicarilla or Juaneno or Acjachemen or Jumano or Kalapuya or Clackama or Kalispel or "Pend d' Oreilles" or Qlispe or Karuk or Karok or "Chum-ne" or Katkoc or Kansa or Kanza or Kawaiisu or Nuwa or Kennebec or "Kinipekw Kittitas" or Klickitat or "Qwu' lh-hwai-pum" or "Awi-adshi" or Mahane or Wahnookt or "Koa' aga' itoka" or Keresan or Kichai or Kitsai or Keechi or "K' itaish" or Kiowa or Gaigwu or Cauigu or Kutjau or "Kwu-da" or "Tep-da" or Kitanemuk or Kittitas or Klickitat or "Qwu' lh-hwai-pum" or "Awi-adshi" or Mahane or Wahnookt or "Koa' aga' itoka" or Konkow or "Koop Ticutta" or Koyukon or Ktunaxa or Kootenai or Flathead or Kucadikadi or "Kotsa' va" or Kumeyaay or "Tipai-Ipai" or Kamia or Diegueno or Kwapa or Cocopah or Cucapa or "Xawitt kwnchawaay" or Lassik or Lenape or "Leni-Lenape" or Lipan or Luiseno or Payomkawichum or Madqwadabaya or "Desert Yavapai" or Mahican or Mohicans or Makah or Makuhadokado or Maliseet or Wolistoqiag or Manahoac or Mahock or Meipontsky or Mandan or Mattole or "Bear River" or "Tul' bush" or "Ni' ekeni" or Meherrin or Menominee or Mackinac or Mescalero or Myaamiaki or Kickapoo or Twigtwee or Missouria or Miwok or Miwuk or Moadokado or Modoc or Mohave or "Aha Makhav" or Mohawk or "Kaneng' hega" or Molala or Molale or Molele or Nyyhmy or Moosonee or "Moose Cree" or Monsonis or Multnomah or Chinook or Nabedache or Nabaydacu or Wawadishe or Nabiltse or Dakubetede or "Nacho Nyak Dun" or Tutchone or Nacono or "Na' isha" or Nanticoke or Navajo or Ndee or Nial or Niimiipu or "Nez Perce" or Watapala or Watapahlute or Nisenan or Nisqually or Nomlaki or Noamlakee or "Central Wintun" or Nongatl or Nottoway or Cheroenhaka or "Northern Cheyenne" or Ohlone or Costanoan or Omaha or "O' odham" or Pima or Papago or Osage or Otoe or Otse or "Ozav Dika" or Palus or Passamaquoddy or Pestomuhkati or Patiri or Petaros or Pastia or Patwin or "Southern Wintun" or Panis or Skidi or Pedee or Penobscot or "Petun Piipaash" or "Kokmalik' op" or Piscatawa or Doeg or Conoy or "Pit River" or Pomo or Kashaya or Ponca or Ponka or Pottawatomi or Bodewadmik or Powhatan or Puyallup or Spuyalepabs or Quapaw or Ugahxpa or Quechan or Yuma or Kwtsaan or Quileute or Salinan or Saponi or Monacan or Sapon or "Eastern Blackfoot" or Christanna or Sawawatodo or Serrano or Taaqtam or "Maarenga' yam" or Yuhaviatam or Shasta or Chasta or Sasti or Shoshone or Siletz or Sinkine or Sinkyone or "Siuslaw Umpqua" or Skitswish or "Schitsu' umash" or Snohomish or Snuqualmi or Sokoki or Missiquoi or Stillaguamish or Stoluckwamish or Suquamish or Sutaio or Swinomish or Skagit or Syilx or Okanagan or Sotaae or "Taga Ticutta" or Takelma or Dagelma or Taltushtuntede or Galice or "Tanan Gwich' in" or Taos or Taovaya or Tataviam or Alliklik or Tawakoni or Tahuacano or Tenino or Thawikila or Hathawekela or "Fort Ancient" or Tigua or Tillamook or Nehalem or Timbisha or Panamint or Timpanogos or Tlingit or "Toi Ticutta" or Tolowa or "Talawa Dini' " or Tongva or Gabrieleno or Fernandeno or Tobikhar or Tonkawa or Ticanwatic or Tsikip or Appalousa or Opelousa or Tsitsistas or Tubatulabal or Tukabatchee or Tuscarora or Tomahittan or Kuskarawock or Tutelo or Tutero or Totteroy or Tutera or Yusan or Tututni or Umatilla or Umpqua or Waccamaw or Waxmaw or Wadatika or "Harney Valley Paiute" or Wailiki or Waluulapam or "Walla Walla" or Walpapi or Huipui or Wampanoag or Massasoit or Wanapum or Wappo or Washoe or Wichita or Willapa or Kwalhioqua or "Wi pukba" or "Verde Valley Yavapai" or Wintu or "Northern Wintun" or Wiyot or "Wee' at" or Weyet or Yakama or "Yamosopo Tuviwarai" or Yaqui or Yoeme or Yatasi or Yattasih or "Yavbe' " or "Yavapai" or "Ysleta del Sur" or Yojuane or Yokuts or Mariposa or Yuki or Yupighyt or Yup'ik or Yupik or Yurok or "Olekwo'l" or Zuni or ((Applegate or Delaware or Iowa or Ishak or Kaw or Kato or Spokane or Miami or Arkansas or Tali or Tunica or Han or Pawnee or "Coeur D' Alene" or Piscataway or Ree or Tula) N3 (reservation* or nation or people or peoples or population or man or men or woman or women or child* or youth* or elder or elders or communit* or tribe or tribes or tribal or Indian* or patient*))) \| 39,761 \| \| S7 \| (Saami or Sampi or (Sami not Ulus) or Samis or Southernsami* or Umesami* or Pitesami* or Lulesami* or Northernsami* or Enaresami* or Kolasami* or Lapp or Lapps or Lappish or Lappland or (Lapland* not longspur) or Lappalainen* or Saamelainen* or reindeer herd* or reindeer culture* or reindeer pastoral* or Lappbys or Samebys or reinbeitesdistrikt or paliskunta or siida) or (((Fennoscandia or Finnmark or Scandinavia or Nordic or Sweden or Norway or Finland or Swedish or Finnish or Norwegian or Norge or Svensk* or Suomi or Barents Region or (Kola not (garcinia or gotu)) or Arctic Europe* or Polar Europe* or North* Europ*)or (MH "Scandinavia") OR (MH "Finland") OR (MH "Norway") OR (MH "Sweden") ) and ((indigenous* or (traditional N3 (food* or heal* or medicine* or shaman*))))) \| 3,823 \| \| S8 \| ((MH "Australia+") or Australia* or "Northern Territory" or "Australian Capital Territory" or Tasmania or "New South Wales" or Victoria or Queensland)) and ( Aborigin* or Indigenous)) or (MH "First Nations of Australia") OR (MH "Aboriginal Australians") OR (MH "Torres Strait Islanders") or Torres Strait Islander* \| 9,288 \| \| S9 \| (MH Maori) or (Maori or "tangata whenua") or (( (MH New Zealand) or (New Zealand or Aukland)) and ( (Aborig* or Indig*))) \| 4,016 \| \| S10 \| TI ( (Acatec OR Aguacateco OR Amuzgo OR Bokota OR Boruca OR Bribri OR "Bri Bri" OR Buglere OR Cabecar OR Cakchiquel OR Changuena or Chatino or Chiapanec OR Chicomuceltec or Chinantee or Chocho OR Cholti or "Ch'olti'" or "Ch'olti'an" or Chontal OR Chorotega OR Chorti OR Chuj or Chumbia OR Corobici OR (Cueva not Spain) or Cuicatec or Cuitlatee or Cuytec OR Dorasque OR Embera OR Garifuna Or Guatuso OR Guaymi OR Guaymis OR Guetar or Huastec or Huave OR Huetar OR Itzaj OR Ixil OR Jacalteco or Jonaz OR Kanjobal OR Kekchi OR Kuna OR Maleku OR Mangue Or Matambu or Matlatzinca or Mazahua or Motozintlec or Mayan OR Mayangna OR Miskito Or Mixtec OR Mopan Or Nahua Or Nahuatl OR Ngabe Or Otomi Or Pantec Or Paya OR Popoloca OR Popoloc OR Poqomam OR Poqomchi OR "Q'eqchi'" OR Quiche OR Quitirrisi OR Sacapulteco OR Sipacapense OR Subtiaba OR Tacaneco OR Tarasco or Tamaulipec or Tamazultec or Tecoxquin OR Tectiteco OR Tecual Or Tecuexe or Tepehura or Tepuztecor OR Teribe OR Terraba OR Totonac or Trique or Tzeltal or Tzotzil OR Tzutujil OR Ulwa OR Uspantec* OR Uspanteko OR Voto or Xinca OR Waunana OR Wounaan OR Yucatec or Zapotec or Zoque) ) AND AB ( (Acatec OR Aguacateco OR Amuzgo OR Bokota OR Boruca OR Bribri OR "Bri Bri" OR Buglere OR Cabecar OR Cakchiquel OR Changuena or Chatino or Chiapanec OR Chicomuceltec or Chinantee or Chocho OR Cholti or "Ch'olti'" or "Ch'olti'an" or Chontal OR Chorotega OR Chorti OR Chuj or Chumbia OR Corobici OR (Cueva not Spain) or Cuicatec or Cuitlatee or Cuytec OR Dorasque OR Embera OR Garifuna Or Guatuso OR Guaymi OR Guaymis OR Guetar or Huastec or Huave OR Huetar OR Itzaj OR Ixil OR Jacalteco or Jonaz OR Kanjobal OR Kekchi OR Kuna OR Maleku OR Mangue Or Matambu or Matlatzinca or Mazahua or Motozintlec or Mayan OR Mayangna OR Miskito Or Mixtec OR Mopan Or Nahua Or Nahuatl OR Ngabe Or Otomi Or Pantec Or Paya OR Popoloca OR Popoloc OR Poqomam OR Poqomchi OR "Q'eqchi'" OR Quiche OR Quitirrisi OR Sacapulteco OR Sipacapense OR Subtiaba OR Tacaneco OR Tarasco or Tamaulipec or Tamazultec or Tecoxquin OR Tectiteco OR Tecual Or Tecuexe or Tepehura or Tepuztecor OR Teribe OR Terraba OR Totonac or Trique or Tzeltal or Tzotzil OR Tzutujil OR Ulwa OR Uspantec* OR Uspanteko OR Voto or Xinca OR Waunana OR Wounaan OR Yucatec or Zapotec or Zoque) ) \| 105 \| \| S11 \| ((MH "Central America") OR (MH "Belize") OR (MH "Costa Rica") OR (MH "El Salvador") OR (MH "Guatemala") OR (MH "Honduras") OR (MH "Nicaragua") OR (MH "Panama") OR (MH "Panama Canal Zone")) and (TI (Indian or Indians or Amerindian* OR Amerindio* or Aboriginal* or Indigenous OR Indigena* or Aborigen* OR Mestizo or tribe or tribes or tribal or "traditional medicine*" or shaman*) or (AB (Indian or Indians or Amerindian* OR Amerindio* or Aboriginal* or Indigenous OR Indigena* or Aborigen* OR Mestizo or tribe or tribes or tribal or "traditional medicine*" or shaman*)) ) \| 208 \| \| S12 \| (TI (Belize or Belizean* or "Costa Rica*" or "El Salvador" or Hondura* or Nicaragua* OR Panama* or Guatemala* or Achi or Maya* OR Mixe OR Pame OR Pipil OR Pech or Chol OR "Ch'olan" or Cora or Cuna or Mam or Rama) N5 (Indian or Indians or Amerindian* OR Amerindio* or Aboriginal* or Indigenous OR Indigena* or Aborigen* OR Mestizo or tribe or tribes or tribal or "traditional medicine*" or shaman*) \| 61 \| \| S13 \| (AB (Belize or Belizean* or "Costa Rica*" or "El Salvador" or Hondura* or Nicaragua* OR Panama* or Guatemala* or Achi or Maya* OR Mixe OR Pame OR Pipil OR Pech or Chol OR "Ch'olan" or Cora or Cuna or Mam or Rama) N5 (Indian or Indians or Amerindian* OR Amerindio* or Aboriginal* or Indigenous OR Indigena* or Aborigen* OR Mestizo or tribe or tribes or tribal or "traditional medicine*" or shaman*)) \| 135 \| \| S14 \| ( ((MH Mexico+) or (Mexico* not "New Mexico*) ) or mexicano or mexicali ) ) AND ( (MH "Indigenous Peoples") or Mesoamerindian* or Indigen* or aborig* or "first people*" or "indos mexicano" or "pueblos indigenas" ) \| 349 \| \| S15 \| ( (MH South American+) or Argentin* or Bolivia* or Brazil* or Chile* or Colombia* or Ecuador* or "French Guiana" or Guyana* or Paraguay* or Peru or Peruvian or Suriname or Uruguay* or Venezuela* or "Amazon Region" or Amazonia or Andes or Andean ) AND ( (MH Native Americans) or (Indian* or Amerindian, or Aboriginal* or indigenas or Indigenous) ) \| 1,179 \| \| S16 \| S1 OR S2 OR S3 OR S4 OR S5 OR S6 OR S7 OR S8 OR S9 OR S10 OR S11 OR S12 OR S13 OR S14 OR S15 \| 69,779 \| \| S17 \| (MH "Community Health Workers") \| 3,985 \| \| S18 \| ( "community health agent*" or "lay health worker*" or "lay health advisor*" or "village health worker* or "local health worker*" or (community n2 "health worker*") or "peer health worker*" ) OR TI CHW AND AB CHW \| 676 \| \| S19 \| S17 OR S18 \| 4,379 \| \| S20 \| S16 AND S19 \| 171 \| |
| --- | --- | --- | --- | --- | --- | --- | --- | --- | --- | --- | --- | --- | --- | --- | --- | --- | --- | --- | --- | --- | --- | --- | --- | --- | --- | --- | --- | --- | --- | --- | --- | --- | --- | --- | --- | --- | --- | --- | --- | --- | --- | --- | --- | --- | --- | --- | --- | --- | --- | --- | --- | --- | --- | --- | --- | --- | --- | --- | --- | --- | --- | --- | --- |

**Global Index Medicus** [**https://www.globalindexmedicus.net/**](https://www.globalindexmedicus.net/) **Searched January 20, 2022**

tw:((tw:((tw:("community health worker*" OR "community health agent*" OR "village health worker*" OR "local health worker*")))) AND (tw:(indigenous OR aborig*))) Result = 35
