# Supplementary material for: Examining global Indigenous community wellness worker models: a rapid review
Source: Int J Equity Health. 2024 May 2;23:90. doi: 10.1186/s12939-024-02185-5 (PMC11065687; doi:10.1186/s12939-024-02185-5)
Supplement: Supplementary file 3 — Supplementary Material 3 [file 12939_2024_2185_MOESM3_ESM.docx]

**Additional File 3: Grey Literature Search Strategy and Terms**

**Methods: Search**

Two searches were conducted using Google and Google Scholar to identify grey literature sources. The former yielded both reports and website content, while the latter generated additional peer-reviewed articles and several reports. Due to the rapid timeframe of the review and the need to quickly mobilize the findings of our research to community partners, we only looked at the first 50 results that were presented in Google and Google Scholar. These results, which were subject to the same criteria as the studies yielded in our academic literature search search, were then screened for inclusion.

| **Date** | **Search engine** | **Search strategy(s) including how items were selected** | **# of items screened** |
| --- | --- | --- | --- |
| March 22nd, 2022 | google.com | 1. "Indigenous mental health worker*" OR "indigenous mental health peer*" OR "Indigenous mental health liaison*" OR "Indigenous mental health helper*" OR "Indigenous mental health agent*" OR "indigenous mental health advocat*"   Selection: Items were selected by scanning the first 50 results | 50 screened  Included:   - Reports and website content (n=4) |
| March 22nd, 2022 | scholar.google.com/ | 1. "Indigenous mental health worker*" OR "indigenous mental health peer*" OR "Indigenous mental health liaison*" OR "Indigenous mental health helper*" OR "Indigenous mental health agent*" OR "indigenous mental health advocat*"   Selection: Items were selected by scanning the first 50 results | 50 screened  Included:   - Academic literature (n=8) - Reports (n=4) |

This template was originally created by Jackie Stapleton and is based on the methods outlined in: Godin K, Stapleton J, Kirkpatrick SI, Hanning RM, Leatherdale ST. Applying systematic review search methods to the grey literature: a case study examining guidelines for school-based breakfast programs in Canada. Systematic reviews. 2015 Dec;4(1):1-0.
