# Supplementary material for: Examining global Indigenous community wellness worker models: a rapid review
Source: Int J Equity Health. 2024 May 2;23:90. doi: 10.1186/s12939-024-02185-5 (PMC11065687; doi:10.1186/s12939-024-02185-5)
Supplement: Supplementary file 4 — Supplementary Material 4 [file 12939_2024_2185_MOESM4_ESM.docx]

**Additional File 4: Data Extraction Tables from Academic and Grey Literature Sources**

| **Table 3** Data extracted from academic literature sources | | | |
| --- | --- | --- | --- |
| **Author(s), Year** | **Research**  **Methods** | **Location** | **Key**  **Findings** |
| Bartik, Dixon and Dart, 2007 (10) | Program evaluation; (mixed-methods) | Australia | Outlined a 3-year program for training Indigenous child and adolescent mental health workers that consists of: employment as a mental health worker with professional support and supervision; a mentoring program; academic study through an accredited program; and a clinical practicum. Authors touch on following considerations for others seeking to implement similar programs: a structured learning environment; management support; ongoing support from supervisors and teachers; and a commitment to personal development. |
| Catts et al., 2013 (32) | Empirical (mixed methods) | Australia | Found that Indigenous sub-teams coordinated within mainstream mental health services represent a best practice model for the delivery of early psychosis interventions to Indigenous peoples. The Sunshine Coast Cultural Healing Program, which follows an Indigenous sub-team model, enhanced the engagement of Indigenous youth. |
| Cleworth, Smith, and Sealey, 2006 (33) | Empirical (quantitative) | Australia | The authors touch on a pilot project that Durri staff and mainstream mental health stakeholders implemented to better support the mental health needs of Indigenous communities in the area. Culturally appropriate methods of engaging Indigenous peoples were identified and the service was found to increase engagement, suggesting the program was valuable. |
| Cosgrave, Maple, and Hussain, 2017 (12) | Empirical (qualitative) | Australia | Factors identified as negatively impacting the job satisfaction of Aboriginal mental health workers include: difficulties integrating into the workplace due to lack of orientation; culturally specific work challenges, like setting personal and professional boundaries; professional differences and inequality when it comes to compensation, as well as reduced career options. |
| de Souza et al., 2015 (35) | Empirical (qualitative) | Brazil | Community health agents play a key role in the identification of mental health needs, and this paper analyzed the perceptions of CHAs regarding mental health care. Authors identified the most frequent mental health care situations  Strengths: living in the community in which they work; use of soft skills, such as establishing relationships with clients.  Difficulties in attending to mental health needs: living in the community in which they work is advantageous, but also presents challenges as it makes it hard for workers to maintain privacy; lack of training in mental health care; emotional burden of work. |
| Dunstan et al., 2014 (39) | Empirical (mixed methods) | Australia | Results show that rural personal helpers and mentors (PhaMs) services are effective in treating mental health. Workforce shortages can be addressed through capacity-building. A few of the PhaMs staff identified as Indigenous, which allowed Indigenous clients to engage with Aboriginal staff. This enhanced participation of Aboriginal clients in mental health services. |
| Etter et al., 2019 (37) | Implementation | Canada | ACCESS OM framework was adapted for use within an Inuit community, which included the training of local AYWs to engage youth in social and cultural activities; provide mental health education; and guide youth to relevant services.  Challenges: AYWs have to manage dual roles (e.g., maintaining confidentiality while also being trusted community members); due to the small size of the community, youth face difficulties in maintaining privacy when seeking help. |
| Harris and Robinson, 2007 (7) | Program evaluation | Australia | An evaluation of the Aboriginal Mental Health Worker Program revealed that the roles of Aboriginal mental health workers are not clearly articulated and require clarification. There is also tension between general practitioners, clinic managers, and AMHWs, who tend to be informally included in clinical work. |
| Hinton and Nagel, 2012 (43) | Empirical (quantitative); Program evaluation | Australia | The Yarning About Mental Health training program, which provides culturally appropriate strategies for understanding and promoting positive mental health, was found to increase knowledge related to Indigenous mental health in the alcohol and other drug workforce. |
| Lauw et al., 2013 (38) | Empirical (qualitative) | Australia | Attributed the success of the Certificate IV qualification course to the following: recognition of personal traumas and abuse in participants; providing the opportunity for participants to touch on prior educational experiences; and restructuring the course to include content on the post-colonization history of Aboriginal peoples. |
| McGuire, 1999 (13) | Theoretical | Australia | Outlined the development of the Certificate IV in Aboriginal Social and Emotional Wellbeing from the perspective of an Aboriginal mental health worker. Challenges faced by AMHWs include: the tendency to take their work home and lack of debriefing opportunities with others who understand the nature of this work. |
| McKenna et al., 2015 (8) | Empirical (mixed methods); case study. | Australia | Aboriginal mental health liaison officers positively influenced the lives of Aboriginal peoples who accessed mental health services, and improved their engagement with other health professionals. Qualitative findings showed that the AMHLO role is multifaceted and facilitates: access to services; understanding among patients and clinicians; discharge pathways and referrals; and continuity of care, as engagement was maintained even after formal discharge. |
| Mushin et al, 2003 (34) | Theoretical | Australia | This article touches on the development of the Koori Kids Mental Health Network, how mainstream and Indigenous workers collaborate to provide culturally appropriate services, and the roles of workers. |
| Nagel and Thompson, 2006 (30) | Empirical (quantitative) | Australia | Changes to service delivery for Indigenous peoples were introduced and AMHWs were hired for the first time. These individuals would work in partnership with a nurse, registrar, and part-time psychiatrist and responsibilities include: providing consultancy services to clinical staff; interpreting Aboriginal terms of reference; supporting assessment; acting as cultural links between patients, families, and service providers; and liaising with other services.  The study showed an increase in the number of patients admitted, which was thought to be due to: an increase in population; more awareness of mental illness in the community; more cases of mental illness; and the hospital becoming more culturally responsive. However, the high readmission rate implied that services still need to be enhanced. |
| Nagel and Thompson, 2010 (42) | Empirical (mixed methods) | Australia | Perspectives of AMHWs were gathered to develop an intervention for Indigenous peoples facing mental health challenges. The intervention was tested in a randomized controlled trial with Indigenous clients; findings show that a stronger focus on the families of clients may support mental health treatment and lead to improved outcomes. |
| Nagel, Thompson, and Spencer, 2009 (47) | Empirical (quantitative)Program evaluation. | Australia | Health care professionals were trained in mental health assessment methods designed for Aboriginal clients. The training was found to be well-received and improved both the knowledge and confidence of these professionals in assessing the mental health needs of clients and providing appropriate courses of treatment. |
| O’Keefe et al., 2021 (11) | Theoretical | United States | This article argues for the employment of IMHWs to improve the state of mental health services in Native American and Alaskan communities. Benefits include enhanced cultural awareness on the part of IMHWs, which can help decrease stigma and other barriers.  Barriers include the different cultural, spiritual, and religious beliefs of community members; and difficulties in separating their professional and personal lives, contributing to burnout. |
| Parker, 2003 (31) | Theoretical | Australia | Outlines challenges facing AMHWs in Australia, such as: a lack of clarity on what constitutes an Indigenous mental health worker, as the term IMHW covers a range of qualifications (i.e., some have PhDs and some are professional social workers or psychologists; the term can also apply to Elders).  Also mention several initiatives supporting this cause (e.g., Charles Sturt University and Torres Strait Islander Health set up a model Aboriginal mental health worker course that provides training all the way from basic certification to a PhD; local Aboriginal-controlled health organizations set up their own educational courses; and the Aboriginal and Torres Strait Islander Health Workforce Strategic Framework which aims to increase the number of IMHWs, clarify their role, etc.) |
| Topp, Edelman, and Taylor, 2018 (24) | Theoretical | Australia | As this is a systematic review, the authors discuss various articles. One program related to mental health is the Aboriginal Mental Health Worker Program, and in this article, the challenges of supporting Indigenous involvement in mental health care in remote communities are outlined. Another article describes factors that impact the job satisfaction of Aboriginal mental health workers in New South Wales.  Barriers outlined in these two studies include: lack of consensus on AHWs role in clinical settings; inequity in career pathways and remuneration as against qualifications and nature of work; role confusion and varied expectations of AHWs; perception among some non-Indigenous providers that AHWs are responsible for ‘anything Aboriginal’; workplace power differentials; role ambiguity and unclear cultural legitimacy source of individual strain and ‘burnout’; tensions relating to service provision to clients with whom there may be family business or personal issues. |
| Waidman, Costa, and da Paiano, 2012 (36) | Empirical (qualitative) | Brazil | Authors tried to identify the perceptions of community health agents regarding mental disorders. Findings include: community health workers have a distorted view of what constitutes mental health issues or challenges (e.g., people with mental disturbance are aggressive, dangerous, etc.) They therefore require more training to develop an understanding of what having a mental disorder really means.  Home visits were perceived as a good strategy of checking in on patients, but the health workers mentioned prioritizing other health concerns over mental wellness as they were deemed more serious or urgent. The authors think that this may be due to mental disorders being invisible. |

| **Table 4** Data extracted from grey literature sources | | | |
| --- | --- | --- | --- |
| **Author(s), Year** | **Source**  **Type** | **Location** | **Key**  **Findings** |
| Aboriginal Resource and Development Services, 2015 (14) | Report | Australia | Non-Indigenous staff should be engaged in cultural training in Indigenous worldviews as they relate to mental health; IMHWs should be involved in the development and delivery of this training; IMHWs should get access to professional development opportunities in Western biomedical approaches to mental health, and receive mentorship from more senior IMHWs; a both-ways model that integrates Western and Indigenous approaches to mental health treatment should be used; staffing levels should be adequate so that if one person takes time off, the others are not left scrambling. |
| Fraser Health, n.d. (41) | Website content | Canada | The Aboriginal mental health liaison program connects Indigenous peoples in British Columbia with culturally appropriate services. This resource outlines the job roles of Aboriginal mental health case managers and liaisons. |
| Goulburn Valley Area Mental Health Service, n.d. (97) | Website content | Australia | Describes the roles of Aboriginal mental health liaison officers, which include support understanding medical procedures and providing external referrals, as well as services provided to Indigenous peoples who use the hospital in Goulburn Valley. |
| NSW Health, 2013 (45) | Report | Australia | Findings show that the NSW Aboriginal Mental Health Worker Training Program increased staff knowledge of Aboriginal mental health; improved the capacity of staff to provide culturally responsive services; and provided local Aboriginal peoples with the opportunity to gain a post-secondary education and qualification as mental health workers. |
| NSW Health, 2014 (46) | Report | Australia | This guide outlines the development of the NSW Aboriginal Mental Health Worker Training Program, its development over the years, and best practice for training AMHWs. |
| Watson and Harrison, 2009 (15) | Report | Australia | Authors evaluated the first year of the NSW Aboriginal Mental Health Worker Training Program to identify practices that could inform future implementation of the program.  Key findings include:  -Briefing support staff about the program and roles of the trainees.  -Preparing an office space for trainees.  -Detailed position descriptions that mention the full-time work and study nature of the job.  -Involving Indigenous communities in the recruitment process.  -Being flexible with criminal record checks.  -Providing thorough orientations for trainees.  -Allocating study days during the week.  -Having realistic expectations about what trainees can do. |
| Robinson and Harris, 2005 (40) | Report | Australia | Outlines findings from the evaluation of the Aboriginal Mental Health Worker Program, which described the work of the AMHWs and assessed the degree to which they were integrated in mental health care. |
| Dudgeon, Milroy, and Walker, 2014 (44) | Report | Australia | This book provides culturally appropriate resources, practices and strategies to enhance the effectiveness of services provided to Aboriginal and Torres Strait Islander peoples dealing with mental health challenges. |
